# An Ichor-dependent apical extracellular matrix regulates seamless tube shape and integrity

**DOI:** 10.1371/journal.pgen.1007146

**Published:** 2018-01-08

**Authors:** Jeffrey B. Rosa, Mark M. Metzstein, Amin S. Ghabrial

**Affiliations:** 1 Department of Cell & Developmental Biology, University of Pennsylvania, Perelman School of Medicine, Philadelphia, Pennsylvania, United States of America; 2 Department of Human Genetics, University of Utah, Salt Lake City, Utah, United States of America; 3 Department of Pathology and Cell Biology, Columbia University Medical Center, New York, New York, United States of America; NYU School of Medicine, UNITED STATES

## Abstract

During sprouting angiogenesis in the vertebrate vascular system, and primary branching in the *Drosophila* tracheal system, specialized tip cells direct branch outgrowth and network formation. When tip cells lumenize, they form subcellular (seamless) tubes. How these seamless tubes are made, shaped and maintained remains poorly understood. Here we characterize a *Drosophila* mutant called *ichor* (*ich*), and show that *ich* is essential for the integrity and shape of seamless tubes in tracheal terminal cells. We find that Ich regulates seamless tubulogenesis via its role in promoting the formation of a mature apical extracellular matrix (aECM) lining the lumen of the seamless tubes. We determined that *ich* encodes a zinc finger protein (CG11966) that acts, as a transcriptional activator required for the expression of multiple aECM factors, including a novel membrane-anchored trypsin protease (CG8213). Thus, the integrity and shape of seamless tubes are regulated by the aECM that lines their lumens.

## Author summary

Biological tubes adopt a variety of shapes to carry out their functions. In addition to multicellular tubes, single epithelial or endothelial cells build unicellular lumens lined by an apical membrane devoid of cell junctions, or seams. Such seamless tubes are highly conserved from invertebrates to vertebrate organs, but the factors regulating their formation and maintenance remain poorly understood in any system. Using a forward genetic approach in the *Drosophila* tracheal (respiratory) system, we have characterized a mutant called *ichor*, which compromises the integrity and shape of seamless tubes in tracheal terminal cells. We demonstrate that Ichor promotes seamless tube integrity and shape by transcriptionally activating genes required to assemble an extracellular matrix (cuticle) lining the lumens of terminal cells. The cuticle has long been thought function as a passive exoskeleton, but this work demonstrates that the cuticle contains signals regulating seamless tube growth and/or maintenance. All tubes are lined by apical extracellular matrices, but their composition, assembly, and functions are poorly understood. By characterizing effectors acting downstream of Ichor, we can systematically identify factors controlling all three processes in a model lumenal matrix.

## Introduction

Biological tubes transport nutrients, respiratory gases, metabolic wastes, and secretions that are essential for viability. To function properly, these tubes must achieve and maintain their proper shapes. Defects in tube shape can result in severe organ malfunction, such as in polycystic kidney and liver diseases, and in hereditary hemorrhagic telangiectasia [1,2].

Most biological tubes are comprised of polarized epithelial or endothelial cells that shape their apical domains to build and expand a tube lumen. To carry out their specialized functions, tubes come in different architectures, shapes and sizes [3]. In multicellular tubes, an epithelial sheet surrounds an extracellular lumenal space with intercellular junctions connecting the cells to each other to form a selectively permeable barrier. Tubes can also be unicellular: an individual cell may wrap around a lumenal space and seal into a tube through the formation of self-junctions (autocellular tube), or may generate an internal subcellular lumenal space unbounded by cell junctions (seamless tube).

Seamless tubes are found across the animal kingdom, from invertebrates to vertebrates. The most extensively characterized seamless tubes are those of the nematode excretory system, the vertebrate vascular system, and the fly respiratory (tracheal) system. Within the duct and canal cells of the *C*. *elegans* excretory system, seamless tubes form by two distinct mechanisms–by fusion of membranes bridged by autocellular junctions, or by fusion of intracellular vacuoles into an internal subcellular tube [4–6]. In the vertebrate vasculature, seamless tubes form within endothelial tip cells that lead the migration of new branches and mediate anastomoses [7–10]. In a striking parallel, tracheal tip cells in *Drosophila* lead outgrowth of primary branches and form seamless tubes that either are blind-ended (terminal cells) or mediate anastomoses (fusion cells) [11]. During embryonic stages, terminal cells will form a single gas-filled seamless tube; however, during larval life terminal cells will emanate dozens of subcellular branches that ramify on internal tissues and organs, with each branch containing a blind-ended seamless tube that serves as the final interface for gas exchange [12]. Despite their ubiquity in the animal kingdom, the mechanisms regulating seamless tube morphogenesis remain poorly understood.

Branching morphogenesis has been examined in a broad range of model systems, with multiple mechanisms implicated in the shaping of tubes [13–34]. Among these are mechanisms in which the apical extracellular matrices (aECMs) that line the tube lumen play a key role. The aECM is a heterogeneous three-dimensional extracellular matrix comprised of polysaccharides, proteoglycans, and glycoproteins. Lumenal matrices are common to tubular organs [35–37] and are thought to regulate multiple steps of lumen morphogenesis; however, their mechanisms of action remain, in general, poorly defined. The lumenal matrix protein GP-135/Podocaylxin is an early marker of apical domains in MDCK cysts [38,39] and is required for epithelial polarity, suggesting a role for lumenal matrix factors in lumen initiation. After apical membranes have been established, they must separate and expand to create a lumenal space. Matrix components are thought to promote lumen expansion by inhibiting adhesion between apical membranes [40,41] and/or creating an extensively glycosylated lumenal surface that promotes the osmotic influx of water to form an expandable gel-like matrix [42–46]. Finally, lumenal matrix components can regulate lumen growth by influencing apical membrane morphogenesis and cell shape [47–53] or by influencing cell rearrangements [52, 54].

Perhaps the most thoroughly studied aECM is that of the multicellular tube (the dorsal trunk) in the embryonic *Drosophila* tracheal system [55]. The most abundant component of insect aECM is chitin [56]. During embryonic development, a transient chitin filament is first deposited within the lumen of the dorsal trunk [57–59]. This filament regulates multiple aspects of tube shape, including length and diameter. In mutants disrupting chitin synthesis, tracheal lumens expand in an uncoordinated manner across the tracheal epithelium, resulting in a cystic lumen that has alternating areas of tube constriction and dilation [57–59]. Later, embryonic tracheal tubes elongate, to create a continuous network, as neighboring tracheal hemisegments fuse together. Coinciding with expansion of the apical domain to accommodate the increase in dorsal trunk tube length, the physicochemical properties of the chitin filament are altered through the action of the chitin deacetylases Vermiform and Serpentine. This covalent modification of chitin filaments is required to restrict axial tube growth [49, 50, 60]–dorsal trunk tubes in mutants lacking both deacytlases become excessively elongated and convoluted. The chitin filament expands in concert with the apical membrane, suggesting the two structures are physically coupled [61]. Through this physical coupling, the chitin filament may create a resistance force counterbalancing axial apical membrane growth [61].

During seamless tube growth, single epithelial cells must dramatically increase their surface area/volume ratio through a dramatic increase in the size of the apical compartment [10, 62], yet the continuity and shape of these lumens must be maintained across this relatively large surface area. How seamless tube diameter and shape are initially determined and then maintained is not known; likewise, the role of the aECM in seamless tube morphogenesis is unknown. The aECM is ideally positioned to coordinate apical membrane morphogenesis along the length of seamless tubes, either by providing mechanical support and by actively influencing apical membrane dynamics. Indeed, during seamless tube elongation in the duct cells of *C*. *elegans*, a transient lumenal matrix maintains lumen integrity by promoting apical membrane expansion [41]. In *Drosophila* terminal cells, the majority of seamless tube growth occurs during larval stages, after the chitin filament has been cleared and replaced by a more mature aECM called the cuticle [63]. Initial cuticle deposition is followed by tracheal gas-filling [64, 65]. The tracheal cuticle is a multi-layered aECM organized into a series of circumferential folds, called taenidia [12, 66]. Our understanding of how aECMs, such as the *Drosophila* cuticle, mediate outside-in signaling in tubular epithelia is greatly hindered by a grossly incomplete understanding of their molecular constituents and their functions. As a result, the outside-in signaling pathways linking aECM components to intracellular regulators of seamless tube morphogenesis remain entirely unknown. Roles for aECMs in tube morphogenesis span multiple organs and tissues from invertebrates to vertebrates, indicating an evolutionarily ancient reliance on aECMs to ensure proper lumen shape. An integrated model for lumen morphogenesis, therefore, requires a better understanding of the molecular components of lumenal matrices and their functions during tubulogenesis.

We find that the tracheal mutant, *ichor* (*ich*), previously identified in a forward genetic screen [67], compromises seamless tube shape and integrity by disrupting the aECM. We determined that *ich* mutations are loss of function alleles of the uncharacterized gene CG11966, which encodes a putative zinc finger transcription factor. Expression of *ich* coincides spatially and temporally with cuticle production, and *ich* is essential for aECM assembly, including of the lumenal matrix lining terminal cell lumens. Further, we show that Ich functions as a transcriptional activator in terminal cells and identify aECM components whose expression is Ich-dependent. Taken together, these data suggest a role for Ich in coordinating the assembly or modification of the aECM, and demonstrate that the aECM plays a crucial role in seamless tube shape and integrity.

## Results

### *ichor (ich)* is required for terminal cell seamless tube shape and integrity

**Fig 1:**
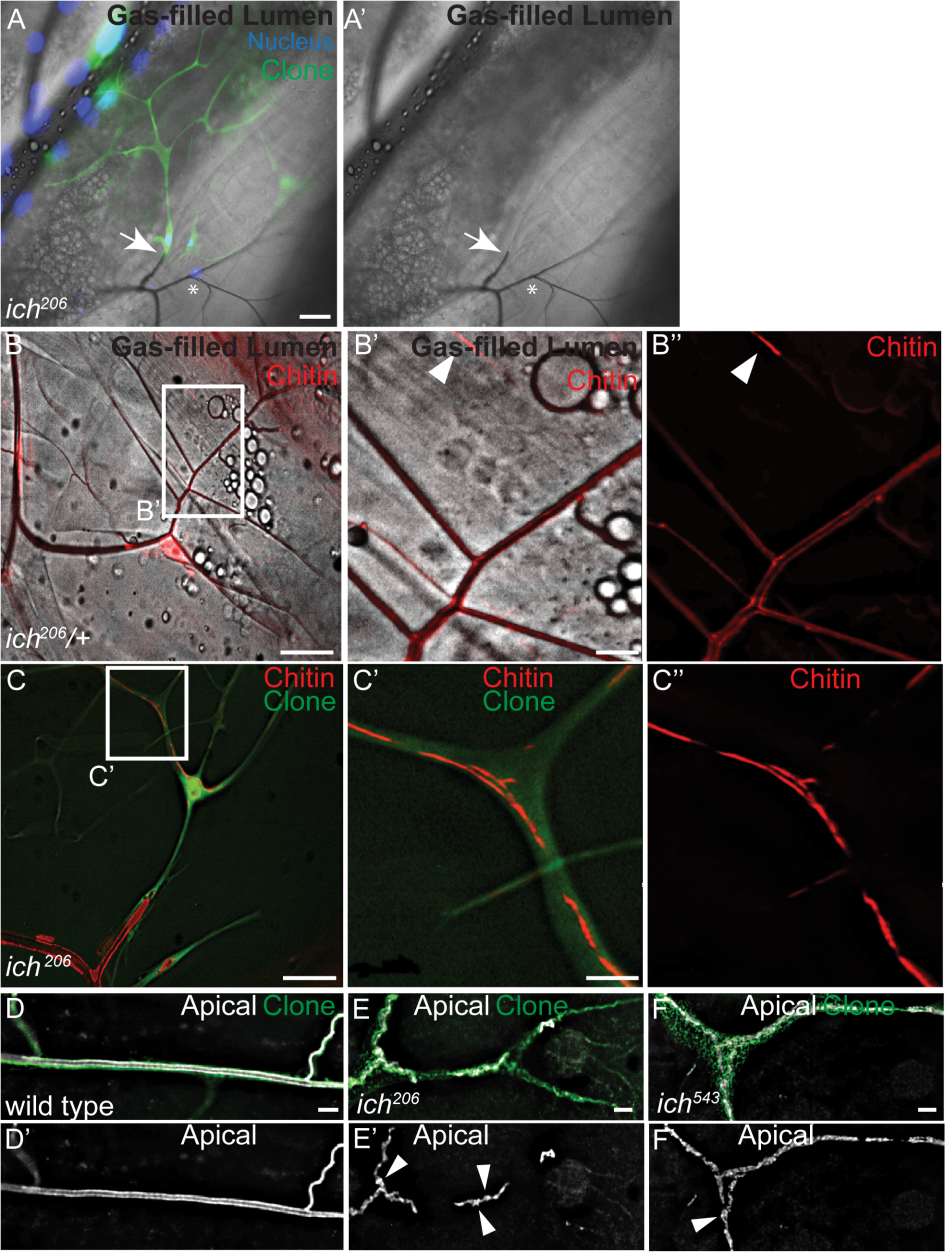
Mutations in *ichor* (*ich*) disrupt seamless tube integrity and shape in larval terminal cells. (A,A’) In a genetic mosaic third instar larva, GFP-labeled *ich*^*206/206*^ terminal cells (green) are visualized next to GFP-negative control terminal cells (*ich*^*206/+*^, asterisk). All tracheal nuclei are labeled (blue). Fluorescent images are superimposed on brightfield. Gas-filled tubes appear as dark lines; the *ich*^*206/206*^ terminal cells lack gas-filled tubes (arrow marks intercellular junction between ich terminal cell and wild type stalk cell). (B-B”) In control terminal cells, ChtVis-TdTomato (red) labels cuticle-lined gas-filled tracheal tubes (B’, B”) as well as fluid-filled lumens found at the tips of terminal branches (arrowheads in B’, B”). In *ich*^*206/206*^ terminal cells (C), ChtVis-TdTomato accumulates in discontinuous, fluid-filled tubes (C’C”). (D-F) The apical membranes in wild-type (D,D’) and *ich* (E-F’) seamless tubes is visualized by an antiserum raised against a Whacked peptide [69]. In contrast to the continuous apical membranes in control (D,D’), *ich* terminal cell tubes display numerous apical membrane discontinuities and regions of cystic apical membrane (arrowheads in E’, F’). (Scale Bars: A, A’, B, and C 50 μm; B’-B”, C’-C” 10 μm; D-F’ 5 μm).

A single mutation in the gene *ichorous*, here renamed *ichor* (*ich*) to avoid confusion with the vertebrate zinc finger transcription factor, *ikaros*, was identified on the basis of a terminal cell-specific gas filling defect (Fig 1A and 1A’, S1E–S1G’ Fig) in a genetic mosaic screen [67]. In contrast to neighboring heterozygous control terminal cells, GFP-labeled terminal cells homozygous for *ich* (see Materials and Methods) lack gas-filled seamless tubes. This phenotype indicates that *ich* terminal cells are defective in some aspect of tube formation and/or liquid clearance. To determine whether *ich* terminal cells lacked lumens, or instead contained fluid-filled lumens, a fluorescent reporter, lum-GFP, was utilized and found to be secreted into a liquid-filled lumenal space [67]. To further characterize the seamless tube defects, we expressed a genetically encoded chitin reporter [68] in heterozygous control cells (*ich*^*206*^*/+)* and GFP-labeled *ich*^*206*^ clones (Fig 1B and 1C”). Third instar larvae were heat-killed and examined in whole-mount preparations. In control terminal branches (Fig 1B–1B”), ChtVis-Tdtomato was observed bounding gas-filled lumens as well as in the fluid-filled tips of terminal branches (Fig 1B’ and 1B”), indicating ChtVisTdTomato labels the cuticle-lined gas-filled lumens as well as fluid-filled lumens. In contrast, ChtVis-Tdtomato in *ich* terminal cells outlines fragmented and discontinuous lumens (Fig 1C–1C”).

Discontinuities were present throughout the terminal cell; for example, in proximal regions connecting the terminal cell to the neighboring stalk cell (Fig 1C), and in distal regions near the growing tips of the cell (Fig 1C”). Staining against a lumenal membrane antigen (anti-Wkd peptide) [69] showed that in contrast to control (*2FRT*) terminal cells, which contained patent tubes with locally uniform morphology (Fig 1D’), *ich* terminal cells exhibited discontinuous membrane-bounded lumens (Fig 1E’ and 1F’) with an irregular cystic appearance. Other defects were noted at a lower penetrance (see S1A, S1A’ and S1D–1D” Fig). Although seamless tubes first form about 13 hours after egg lay (ael) [11], late stage *ich* embryos (16 hrs ael and older, S1B and S1C Fig) did not exhibit defects in initial seamless tube lumen extension, indicating that the tube discontinuities must arise after embryogenesis is complete. Collectively, these data suggest that *ich* disrupts multiple aspects of tube morphogenesis in terminal cells, including seamless tube growth, shape, and integrity.

### *ichor* encodes an uncharacterized zinc finger protein

**Fig 2:**
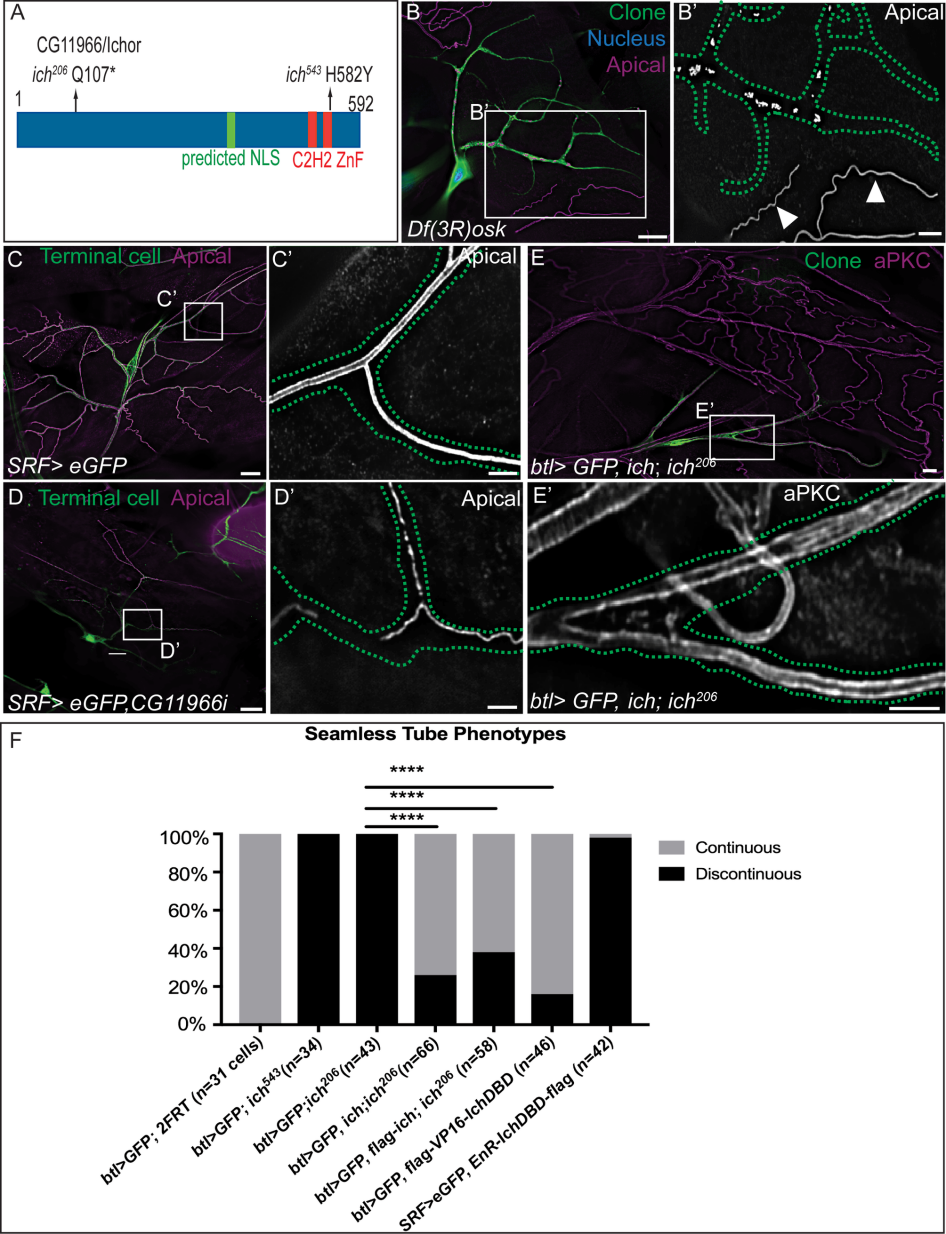
*ichor* encodes a zinc finger protein. (A) Domain map of Ichor polypeptide and position of EMS-induced lesions. (B-B’) In contrast to internal control (*Df(3R)osk/+*) seamless tubes (arrowheads in B’), GFP-labeled *Df(3R)osk* terminal cell clones exhibit *ich-*like apical membrane discontinuities. Apical membrane in B-D is detected using anti-Wkdpep sera. Outline of Df(3R)osk/Df(3R)osk terminal cell shown as green dotted line. (C-D’) *SRF>eGFP* wild-type control terminal cells exhibit a patent seamless tube (C-C’), whereas *SRF>eGFP*, *CG11966* RNAi terminal cells (D-D’) contain discontinuous blind-ended tube fragments. Terminal cell outline shown as green dotted line. (E-F) Restoring expression of full-length CG11966 cDNA in *ich* terminal cells rescues the *ich*^*206*^ discontinuous tube defect, as observed by visualizing apical membranes with aPKC staining (E, E’). (F) Quantification of rescue experiments using Ich transgenes (*P* = 8.82 x 10^−17^ for UAS-*ich*, *P =* 1.83 x 10^−12^ for UAS-flag-*ich*, *P* = 2.01 x 10^−18^, using one-sided Fisher’s exact probability test). (Scale Bars: B,C,D,E 20μm; B’, C’,D’, E’ 5 μm).

From the original screen [67], only a single allele of *ich* (*ich*^*206*^) was isolated. To facilitate mapping and gene characterization, an EMS mutagenesis and non-complementation screen was performed to recover additional *ich* alleles (see Materials and Methods). We recovered a single new allele (*ich*^*543*^*)* that exhibited identical terminal cell defects (Fig 1F–1F’, Fig 2F). Genetically, *ich*^*206*^ and *ich*^*543*^ behaved as strongly hypomorphic or amorphic alleles (see Materials and Methods); both were recessive embryonic lethal, and indistinguishable in homozygous and hemizygous conditions (S1J Fig). Using standard procedures (see Materials and Methods) we mapped *ich* to CG11966 (Fig 2A). The CG11966 open reading frame encodes a 592 amino acid protein with two C2H2-type zinc fingers and a predicted nuclear localization signal (NLS) (G473-Q483). The *ich*^*206*^ allele carries a non-sense mutation (coding for Q107→Stop) upstream of the two C-terminal zinc fingers, while the *ich*^*543*^ allele carries a missense mutation (coding for a H582→Y) resulting in the substitution of a tyrosine residue for a histidine that is required for zinc ion coordination in the second zinc finger. Combined with our genetic analysis, this suggests that at least the second zinc finger domain is essential for Ich function. Further establishing the identity CG11966 as *ich*, we showed that terminal cells homozygous for a small chromosomal deletion [70] uncovering CG11966 (as well as *oskar*, *skap*, and CG11964) (Fig 2B’B’) show the *ich* phenotype. Likewise, expression of a dsRNA (hereafter *ich* RNAi) targeting the CG11966 transcript induced terminal cell-specific tube discontinuities (Fig 2C– 2D’). Finally, full-length and flag-tagged *ich* cDNAs were used to generate transgenic strains (see Materials and Methods), and expression of wild type CG11966 under control of pan-tracheal *btl*-GAL4 [71] was able to rescue seamless tube continuity in ~67% of *ich* mutant clones (Fig 2E and 2F). Interestingly, expression level-dependent gain of function defects in morphology, including terminal cell pruning (Fig 2E and S2C–S2D’ Fig), were also observed.

### Ichor is essential for the generation of mature aECM

To better understand *ich* function during tracheal development, we sought to characterize its expression pattern during embryogenesis. Though our genetic mosaic analysis indicated *ich* is required cell-autonomously in the trachea, previously published RNA *in situ* data [72] do not show *ich* expression in the trachea, suggesting *ich* may be weakly expressed. We used a LacZ enhancer trap, *P{PZ}l(3)05652*, at the *ich* locus to better characterize *ich* expression in the trachea and other tissues. From 9–15 h ael, the *ich*::*nLacZ* transcriptional reporter was expressed specifically in ectodermally-derived epithelia, including the trachea, that secrete cuticle (S3 Fig). This expression pattern was consistent with a role for Ich in aECM assembly and/or modification, which was further supported by the observation that *ich* transcripts are expressed during cuticle deposition in the pupal wing epithelium [73].

**Fig 3:**
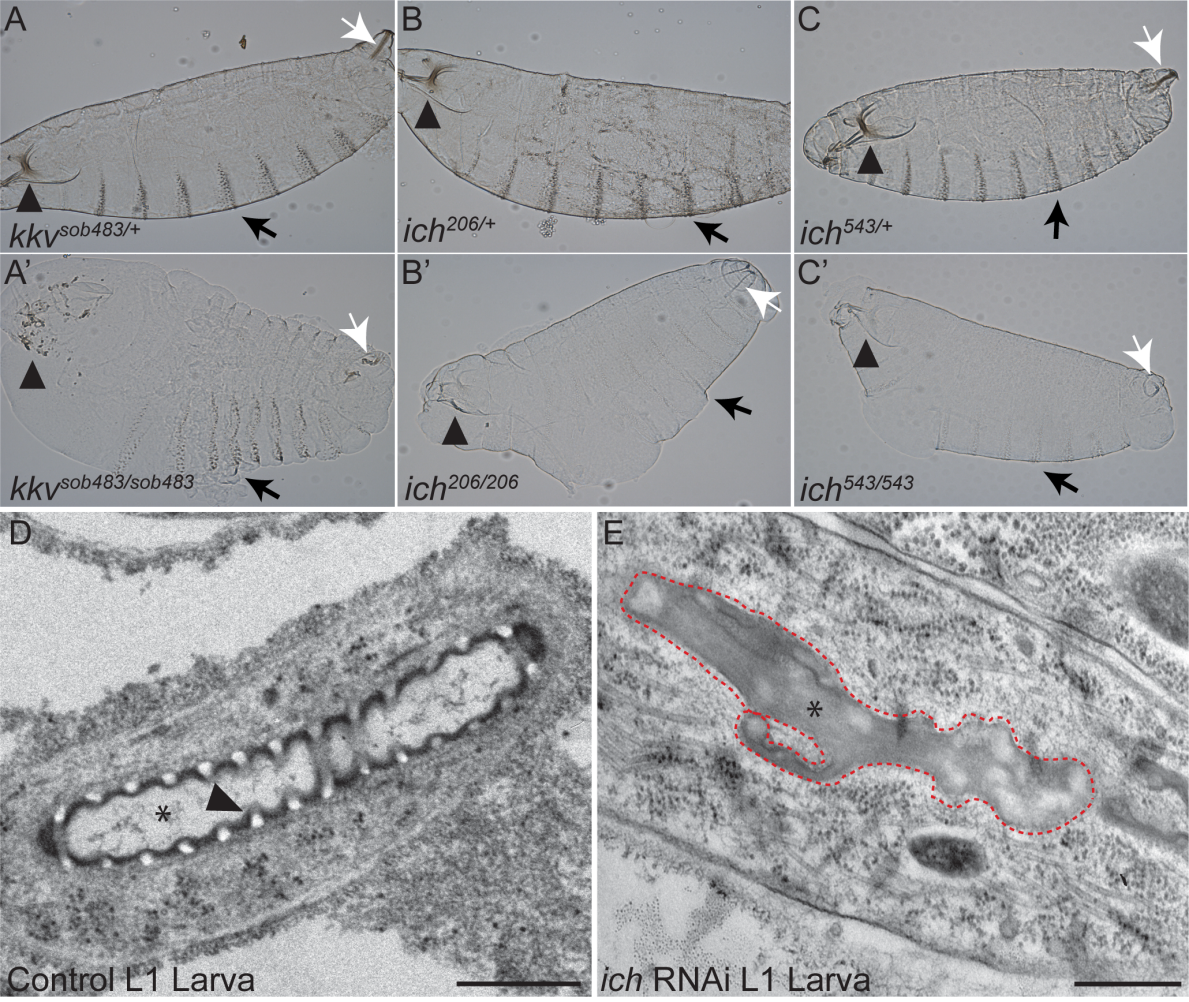
Ichor is required for assembly of the mature aECM. Cuticle preparations of heterozygous control (A-C) and homozygous mutant *kkv*^*sob483*^ (A’), *ich*^*206*^ (B’), and *ich*^*543*^ (C’) St.17 embryos or first instar larvae. Chitin-deficient embryos (A’) exhibited a bloated cuticle and degenerated head skeleton (arrowhead in A’). The *ich* embryos exhibited a mild bloated phenotype as well as cuticle defects distinct from chitin-deficient embryos. The head skeleton (arrowheads in B’, C’) appeared intact but poorly pigmented. Other cuticular structures, such as the epidermal denticles (arrows in B’, C’) and spiracular chambers (white arrows in B’, C’), were also poorly pigmented in *ich* mutants. By contrast, chitin synthesis is not absolutely required for pigmentation of the head skeleton remnants (arrowhead in A’), denticle belts (black arrow in A’), or spiracular chambers (white arrow in A’). (D, E) Longitudinal sections of control (*btl>GFP*, D) and ich RNAi (*SRF>eGFP*, *ich* RNAi; E) first instar (L1) terminal cell tubes visualized by transmission electron microscopy (TEM). Control terminal cell branches contain tubes (*) of locally uniform dimensions lined by an electron-dense aECM (cuticle). The arrowhead in D points to a clearly defined taenidium with an electron-luscent chitinous core. By contrast, the lumens (*) of *ich* RNAi terminal branches adopt a highly irregular morphology (red dashed line). These lumens are devoid of taenidiae and instead are occluded with disorganized electron-dense material. (Scale Bars: D, E = 500 nm).

To investigate further, we asked whether *ich* embryos, like embryos with known defects in the production of a mature aECM (cuticle), would appear grossly inflated in cuticle preparations–the “blimp” phenotype, which reflects an increased elasticity (compare Fig 3A and 3A’) [74]. Both *ich*^*206*^ and *ich*^*543*^ embryos exhibited mild “blimp” defects in cuticle preps (Fig 3B’) as well as reduced sclerotization of the head skeleton and ventral epidermal denticles (Fig 3B’ and 3C’), which are also characteristic of aECM defects. These defects were milder than those observed in embryos mutant for chitin synthase, suggesting that *ich* embryos are not completely chitin deficient.

In the tracheal system, a chitin filament is generated prior to the mature cuticle, and plays an essential role in tube expansion [57–59]. We find that the production of the chitin filament is unaffected in *ich* embryos (S4 Fig), consistent with *ich* not being required for chitin synthesis.

Though Ich is dispensable for tracheal specification, branching, and lumen growth during embryogenesis, pan-tracheal depletion causes a liquid-clearance defect and breaks in the multicellular dorsal trunk tubes in first instar larvae (S1H and S1I’ Fig). Since tracheal cuticle assembly is required for gas-filling [64] and tracheal tube integrity [12,75], we next visualized the ultrastructure of the tracheal cuticle in larval terminal cells using transmission electron microscopy (TEM). Wild-type terminal branches have locally uniform lumens lined by a cuticle organized into taenidia (Fig 3D). By contrast, terminal branches in *ich*-depleted cells have irregularly-shaped lumens devoid of taenidia and instead occluded with electron-dense material (Fig 3E). The electron-dense material may represent mis-assembled matrix material. These phenotypes indicate that Ich is required for lumenal matrix assembly in seamless tubes and suggest that seamless tube shape requires a properly assembled lumenal matrix. Insofar as Ich is dispensable for chitin filament formation, we suggest that Ich functions in the trachea as a specific regulator of the mature aECM (cuticle).

### Ichor functions as a transcriptional activator

**Fig 4:**
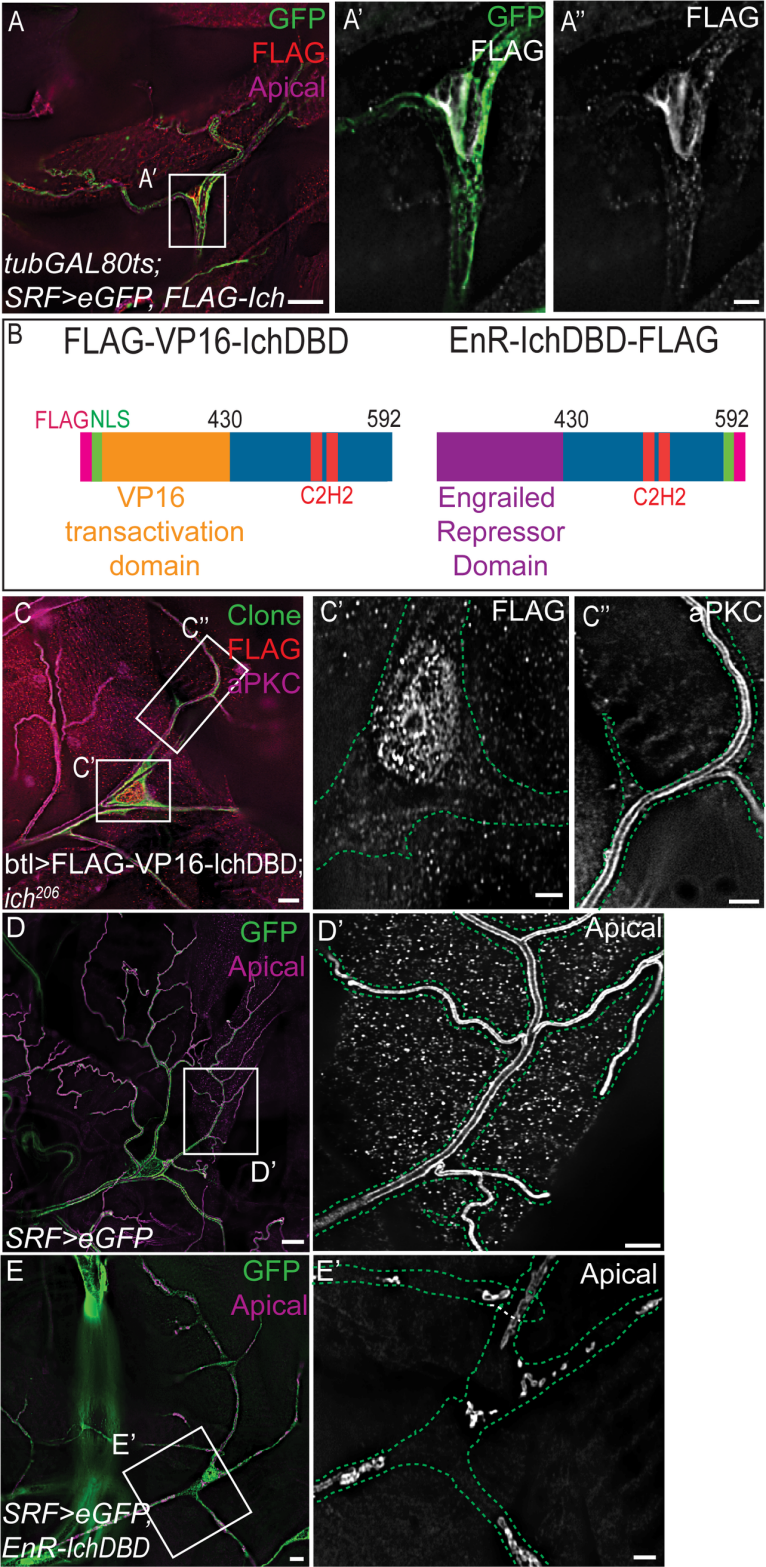
Ichor functions as a transcriptional activator in terminal cells. (A-A”) *tubGAL80*^*ts*^*; SRF>eGFP*, *FLAG-Ich* larvae were upshifted to the restrictive temperature around the start of the third larval instar to induce GAL4-dependent FLAG-Ich expression. FLAG-Ich accumulates at steady-state in the nucleus of terminal cells (A’, A”). (B) Domain organization of FLAG-VP16AD-IchDBD and EnR-IchDBD-FLAG chimeras is schematized. VP16AD (orange) is a potent transcriptional transactivating domain from a viral protein, while EnR (purple) is the transcriptional repressor domain of the Engrailed transcription factor. IchDBD (blue) is the C-terminal portion of the Ich protein that includes the zinc finger domains (red) presumed to confer DNA binding. (C-C”) *ich*^*206*^ MARCM terminal cell clones expressing UAS-FLAG-VP16AD-IchDBD transgene. By virtue of an exogenous nuclear targeting sequence (see B, green), FLAG-VP16AD-IchDBD chimera accumulates in the nucleus (C, C’), restoring seamless tube integrity (C”), as observed by staining for the apical marker aPKC. (D-E’) Wild-type control *SRF>eGFP* (D-D’) and *SRF>eGFP*, *EnR-IchDBD-FLAG* (E, E’) terminal cells. The EnR-IchDBD-FLAG chimera behaves like an Ich dominant negative, inducing the *ich* loss of function phenotype. In contrast to the fully patent lumens of control terminal cells (D’), *SRF>eGFP*, *EnR-IchDBD-FLAG* terminal cells exhibited blind-ended, discontinuous lumens similar to *ich* mutant terminal cells. (Scale Bars: A, C, D, E 20 μm; A’ and A”, C’ and C”, D’, E’ 5 μm).

Since the *ich* locus had not been previously characterized, we next sought to address Ich molecular function. To test the hypothesis that Ich acts as a transcription factor, we first examined the subcellular localization of a functional (Fig 2E) Flag-tagged isoform of Ich. UAS-Flag-Ich was expressed in terminal cells using the GAL4/UAS two-component system [76]. To limit over-expression phenotypes, we utilized a *tubGAL80*^*ts*^ transgene [77] and shifted temperature to permit Flag-Ich expression only in third instar larvae (Fig 4A–4A”). As predicted for a transcription factor, Ich localizes at steady-state to terminal cell nuclei (Fig 4A’ and 4A”). To test whether Ich functions as a transcriptional activator, we assayed the rescuing activity of an Ich-VP16 chimera. The chimeric protein consisted of the putative DNA binding domain of Ich (the Ich zinc fingers) fused to the transcriptional activation domain of the viral protein VP16 [78] (Flag-VP16-IchDBD, Fig 4B). An exogenous NLS was introduced to ensure nuclear localization of the Flag-VP16- IchDBD chimera (Fig 4C’). Expression of Flag-VP16-IchDBD in *ich*^206^ terminal cells restored seamless tube continuity in 84% of cells (n = 46 cells) (Fig 4C”, Fig 2F). This result indicates that the essential terminal cell function of Ich is as a transcriptional activator. Consistent with this hypothesis, a fusion of the GAL4 DNA binding domain to full-length Ich (GAL4DBD-Ich) induced transcriptional activation of an artificial UAS-Luciferase reporter construct in S2 cells [79]. If Ich functions primarily as a transcriptional activator, then we predicted that ectopic recruitment of repressive machinery to Ich target genes in terminal cells would phenocopy *ich* loss-of-function. We expressed a chimeric Ich consisting of the Engrailed transcriptional repressor domain [80] fused to the Ich zinc fingers (EnR- IchDBD-Flag, Fig 4B), and found that EnR- IchDBD-Flag localized to the nucleus of terminal cells (S2B and S2B’ Fig) and induced *ich*-like tube discontinuities in 98% of cells (n = 42 terminal cells) (compare Fig 4D and 4D’ with Fig 4E and 4E’; Fig 2F). Importantly, the phenotypes caused by overexpression of EnR- IchDBD-Flag are distinct from those caused by overexpression of full-length Ich (see S2 Fig).

### A requirement for chitin in seamless tubes

**Fig 5:**
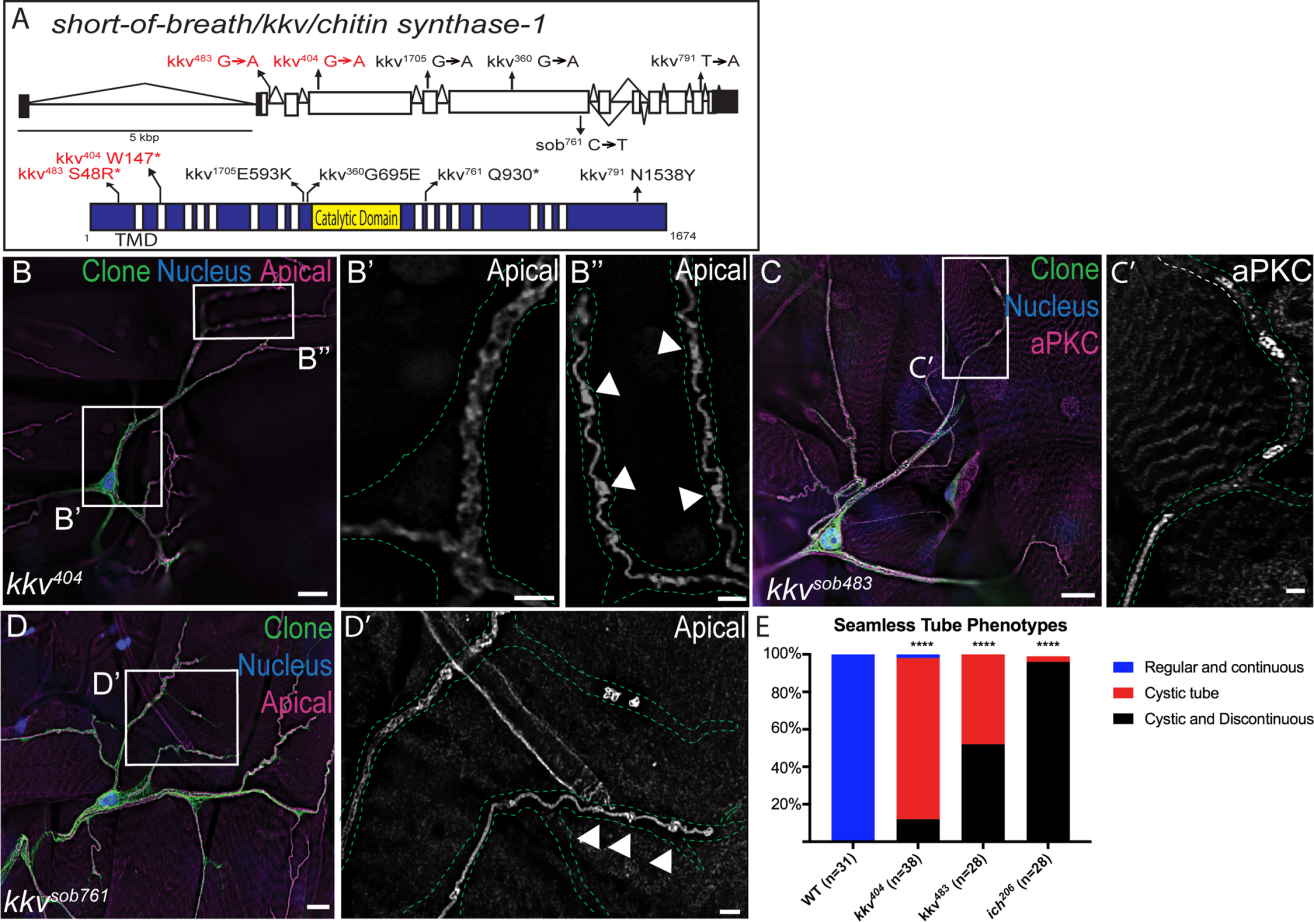
The aECM component chitin is essential for seamless tube shape and integrity. (A) Schematic of the *kkv* gene and the encoded Chitin Synthase protein structure with the position of the *sob* mutations indicated. (B-B”) *kkv*^*sob404*^ null terminal cell clone (green) exhibited apical membranes with cystic, irregular morphology. For the areas marqueed in white, the apical membrane is shown enlarged in (B’, B”) as indicated. Terminal cell outlines in B’, B”, C‘, D’ are shown by green dots. In (C) and (D), *kkv*^*sob483*^ and *kkv*^*sob761*^ clones, respectively, are shown. In distal terminal branches (B”, D ‘), discrete apical membrane cysts (arrowheads) are interspersed with tube of apparently normal morphology. (C-D’) limited regions of discontinuous apical membrane (C’,D’) were also observed with all *kkv* alleles. (E) Penetrance of tube phenotypes for the *kkv*^*sob404*^ null allele and *kkv*^*sob483*^, a putative recessive antimorphic allele (*P*<0.0001, Chi-Square test). (Scale Bars: B, C, D 20 μm; B’,B”, C’, D’ 5 μm).

The best-characterized component of the insect aECM is chitin [66, 74, 81–83]; however, its requirement in seamless tubes has not been directly examined. While ich is not required for the chitin filament in embryonic development, we wished to determine if the *ich* seamless tube defects in terminal cells derive from the loss of chitin in the mature aECM. We turned to mutations in *kkv*, which encodes the sole tracheal chitin synthase. The *short-of-breath (sob)* alleles of *kkv*, were identified by their gas-filling defects (S5A and S5A’ Fig) [67], but had not been more thoroughly examined. We now report that the transient chitin filament normally present in the embryonic tracheal lumens cannot be detected in *kkv*^*sob404*^ and *kkv*^*sob483*^ embryos (S5D–S5G’ Fig), which displayed the cystic dorsal trunk defect (S5H–S5L Fig) characteristic of *kkv*^*1*^ embryos [57, 58]. All 6 *sob* alleles carry point mutations predicted to disrupt the *kkv* coding sequence (Fig 5A) and two of these seemed likely to be null for *kkv* function. The *kkv*^*sob404*^ allele carries a non-sense mutation predicted to truncate the open reading frame after 147 amino acids (Fig 5A) and the *kkv*^*sob483*^ allele disrupts a conserved nucleotide in the splice donor site just upstream of the second intron, predicted to result in a S48→R missense mutation followed immediately by an in-frame stop codon. By genetic assays (S5H–S5L Fig, see Materials and Methods), *kkv*^*sob404*^ behaved as a strong hypomorphic or null allele, whereas *kkv*^*sob483*^ exhibited recessive antimorphic properties (or else carries a second-site modifier).

If a loss of chitin synthesis underlies the *ich* tube defects in terminal cells, then chitin-deficient terminal cells should phenocopy *ich* mutants. The apical membrane in *kkv*^*sob*^ terminal cells was cystic (cysts marked by arrowheads), showing irregular contours (Fig 5B, 5B” and 5C’), in contrast to the smooth, locally uniform apical membrane of control terminal branches (Fig 1D and 1D’). In addition, *kkv* terminal branches contained apical membrane discontinuities, albeit with limited penetrance and expressivity (Fig 5C’ and 5D’). The penetrance of these discontinuities varied with the *kkv*^*sob*^ allele used (Fig 5E): whereas *kkv*^*sob404*^ terminal cell clones exhibit discontinuities in ~12% of terminal cells examined (n = 38), *kkv*^*sob483*^ clones exhibit discontinuities in ~52% of terminal cells examined (n = 28). This difference in penetrance is consistent with the analysis of allele strengths described above (S5H–S5L Fig). Additionally, while multiple terminal branches per cell showed discontinuities in *ich* terminal cells, discontinuities in *kkv*^*sob*^ terminal branches were typically limited to a single branch per cell. We conclude from these studies that a chitin-based aECM is required for seamless tube shape, and that it also contributes to tube integrity while not being absolutely essential. Interestingly, this suggests that other Ich-dependent aECM components may play chitin-independent roles in the maintenance of tube integrity.

### Ich regulates expression of factors important for mature aECM assembly

Since the transcriptional targets of Ich regulation are not known, we sought to identify candidate genes. To focus our search, we utilized two previously published RNAseq data sets, one from the modENCODE consortium [84] and one from the Adler lab, which generated a systematic RNAseq data set across the stages of cuticle deposition in the pupal wing [73]. We reasoned that *ich* target genes would be co-expressed with *ich*, and might be enriched in the overlap of the *ich* co-expression clusters from each data set. We manually selected 4 genes, each expressed in the embryonic trachea, to examine for *ich*-dependent expression: *ectodermal (ect)*, *osi18*, *osi19*, and *CG8213*.

Ect is an apically secreted protein expressed exclusively in chitin-secreting epithelia (S6A–S6D Fig) [85]. Depletion of *ect* in the pupal wing epithelium causes defects in cuticle assembly [73], suggesting Ect is a structural component of chitin-based cuticles. Although *ich* is dispensable for tracheal expression of *ect* (S6B and S6C–S6F Fig), *ich* is required for full *ect* expression in the foregut primordium and the epidermis.

**Fig 6:**
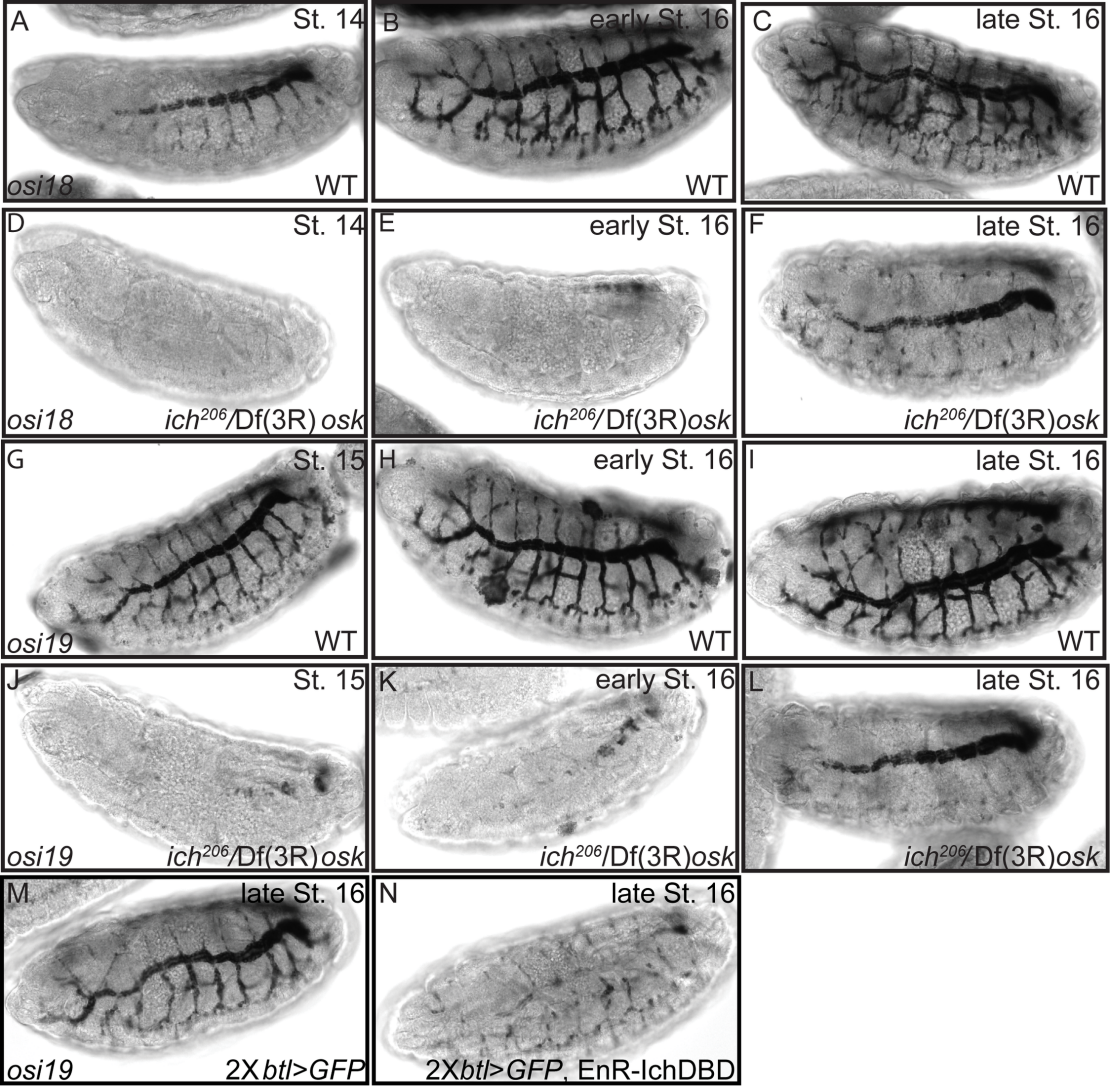
Ichor is required for the pan-tracheal expression of *osi18* and *osi19*. Expression of *osi18* mRNA (A-F) was examined in sibling (WT; A-C) and *ich*^*206*^/Df(3R)*osk* embryos (D-F) by in situ hybridization. Expression of *osi19* mRNA (G-N) was examined in siblings (G-I) and *ich*^*206*^/Df(3R)*osk* embryos (J-L) by in situ hybridization. In contrast to the wild type control heterozygous siblings (A-C and G-I), *ich*^*206*^ hemizygotes (D-F, J-L) lacked *osi18* and *osi19* expression throughout the trachea. However, by late Stage 16 (F, L) *osi18* and *osi19* mRNA became detectable in the multicellular dorsal trunks branches of the tracheal system. Stage 16 control (2X*btl>GFP*) embryos (M) detected the expected pattern of *osi19* mRNA expression in contrast to embryos (N) expressing a transcriptional repressor domain (EnR) fused to the Ich DNA binding domain (IchDBD). The embryos expressing the Ich chimera (2X*btl>GFP*, EnR-IchDBD) exhibited pan-tracheal reduction of osi19 mRNA.

Like all Osiris family members (24 in total), Osi18 and Osi19 are small proteins with predicted signal peptides and transmembrane domains [86]. Gene expression studies suggest Osiris family members may play a role in aECM assembly [73, 87], perhaps by affecting one or more steps of membrane trafficking [88]. Expression of *osi18/19* mRNA initiates ~10h ael specifically in the trachea. Consistent with a role for Osi18/19 in tracheal aECM maturation, *osi18/19* expression is coincident with aECM secretion and modification (S7 Fig). Wild-type *ich* function is essential for the expression of both *osi18* and *osi19*. Embryos mutant for *ich* (*ich*^*206*^*/Df(3R)osk)* exhibit a delay in the onset of *osi18/osi19* expression in the trachea (compare Fig 6A with 6D, 6B with 6E, Fig 6G with 6J and 6H with 6K). By ~15h ael *osi18* and *osi19* mRNA are readily detected in the dorsal trunks of *ich*^*206*^*/Df(3R)osk* embryos (Fig 6F and 6L) although expression in other tracheal branches remains severely reduced as compared to control siblings (compare Fig 6C with 6F and 6I with 6L). That *osi18* and *osi19* expression in the dorsal trunks eventually becomes strong in the absence of *ich*, suggests that other transcriptional regulators expressed in the dorsal trunk can compensate. Consistent with this hypothesis, recruitment of repressive transcriptional machinery to *ich*-regulated loci through the tracheal expression of EnR-IchDBD-Flag confers a complete loss of dorsal trunk *osi19* expression (compare Fig 6N with 6M), suggesting that Ich is part of a network of transcription factors controlling gene expression during maturation of the tracheal aECM.

**Fig 7:**
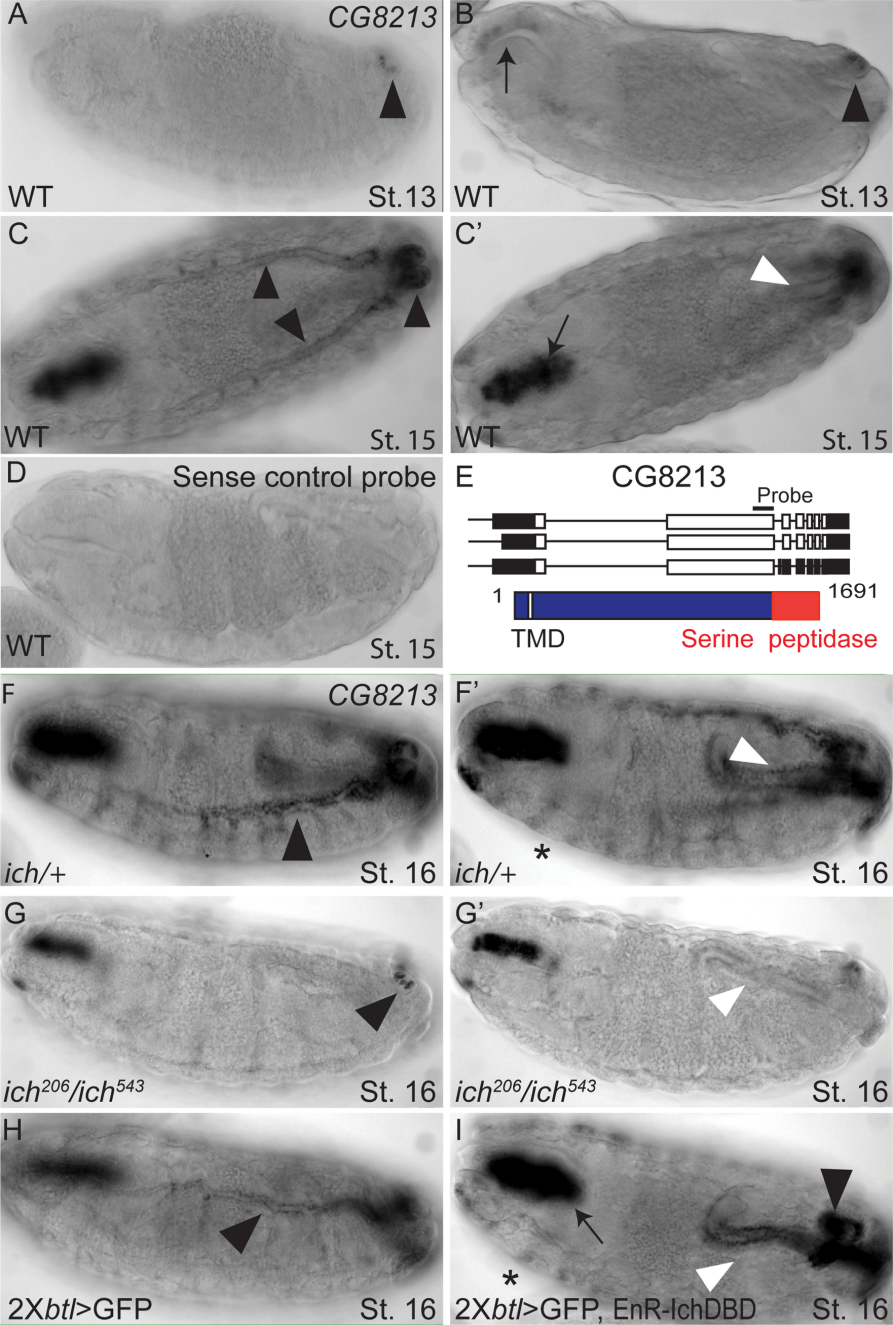
**Ichor is required for CG8213 expression in the trachea** (A-D) Wild-type *w*^*1118*^ embryos hybridized with DIG-labeled antisense (A-C’) and sense (D) *CG8213* RNA probes. CG8213 expression is first detected at St. 13 in the posterior spiracles (arrowhead in A, A’) and foregut primordium (arrow in B). By St. 15, CG8213 is expressed specifically in cuticle-secreting epithelia, including the trachea (black arrowheads in C), foregut (arrow in C’), hindgut (white arrowhead in C’), and epidermis (see asterisks in F’, I). This signal is specific since a sense probe (D) shows no such pattern. (E) Transcripts and polypeptide encoded by CG8213 locus. Black bar denotes position of RNA probes, designed to detect all spliceoforms. (F-G’) *ich*^*206*^*/ich*^*543*^ mutant (G, G’) and control siblings (F,F’) hybridized with antisense CG8213 RNA probe. Whereas control siblings show a WT *CG8213* expression pattern, *ich* mutant embryos exhibited a significant reduction or loss of CG8213 expression in the trachea. Only a subset of cells in the posterior spiracles (black arrowhead in G) retain *CG8213* expression. Hindgut expression (white arrowhead in G’) is also reduced. (H-I) 2X*btl>GFP* (H) and 2X*btl>GFP*, *EnR-IchDBD* embryos hybridized with antisense *CG8213* probe. Whereas control embryos (H) expressed *CG8213* prominently in the major dorsal trunk (black arrowhead in H) of the trachea, tracheal-autonomous expression of the dominant-negative Ich transgene caused a loss of tracheal expression; only cells in the posterior spiracles retain *CG8213* message, similar to *ich* loss-of-function phenotype. *CG8213* expression in the epidermis (asterisk in I), foregut (arrow in I), and hindgut (white arrowhead in I) is unaffected, indicating a tracheal-autonomous loss of expression.

The *ich* candidate target gene, CG8213, encodes a transmembrane protease. RNAi studies in the pupal wing epithelium implicate CG8213 in cuticle assembly [73]. The expression pattern of CG8213 during embryogenesis is highly reminiscent of *ich* (Fig 7A–7D). To determine whether Ich is required for *CG8213* expression in embryonic trachea, *ich*^*206*^*/ich*^*543*^ embryos and sibling controls (*ich*^*206*^ or *ich*^*543*^*/+*) were examined by *in situ* hybridization. While sibling controls (Fig 7F and 7F’) showed the expected wild type pattern, *ich*^*206*^*/ich*^*543*^ embryos showed a loss of both pan-tracheal (arrowhead Fig 7G) and hindgut (arrowhead Fig 7G’) expression of *CG8213*. Foregut expression (Fig 7G’) of *CG8213* persisted in *ich* mutant embryos, and thus is independent of Ich function and also serves as a convenient internal control. Tracheal expression of *CG8213* in *ich*^*206*^*/ich*^*543*^ embryos was restricted to a small number of cells at the posterior spiracle, indicating that Ich is required (either directly or indirectly) for the induction of *CG8213* expression in the trachea.

As an independent confirmation of this result, we found that pantracheal expression of the dominant negative Ich transgene (EnR-IchDBD-Flag) produced a tissue-autonomous reduction in CG8213 expression. Indeed, while control embryos showed prominent tracheal expression of *CG8213* by ~11h ael (arrowhead, Fig 7H), embryos with pan-tracheal expression of EnR-IchDBD-Flag showed a loss of *CG8213* transcript exclusively in the trachea. As in *ich*^206^/*ich*^*543*^ mutants, tracheal expression of *CG8213* persisted only in the posterior spiracles (arrowhead, Fig 7I).

**Fig 8:**
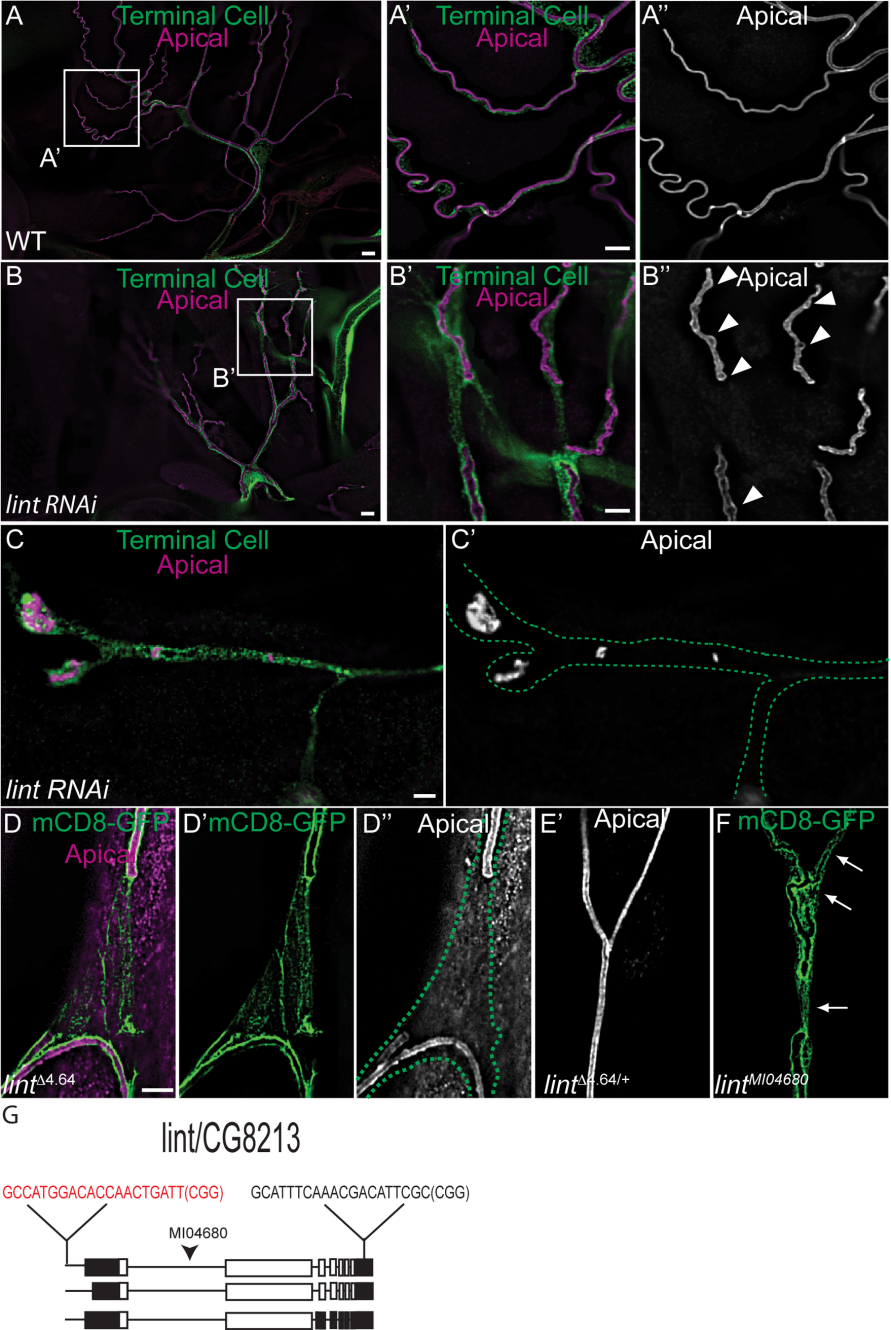
*lumens interrupted* (*lint*) is required for seamless tube integrity in terminal cells. Wild type (WT, *SRF>eGFP*) control (A-A”) and short hairpin RNA against CG8213 expressing terminal cells (B-B”) were examined for apical membrane integrity. In contrast to control terminal branch seamless tubes (A’, A”), seamless tubes in lint-depleted terminal cells exhibit cystic dilations (arrowheads in B”), and discontinuities of the apical membrane with 100% penetrance (n = 33 terminal cells) (B’.B”). The most severely affected *SRF>eGFP*,*CG8213* RNAi terminal cells are severely pruned with terminal branches largely devoid of luminal membrane, except for isolated inclusions. Terminal cell outlines are shown by green dots (C,C’). (D-D”) Details of *lint*^*Δ4*.*64*^ seamless tube defect from a mCD8-GFP-expressing terminal cell clone. The outline of the terminal cell is indicated by green dots. The seamless tubes of a neighboring heterozygous terminal cell (mCD8-GFP negative) are shown in (E’). The gRNA sequences used to target lumens interrupted (lint) using CRISPR/Cas9 are indicated in (G) on a schematic depicting lint gene organization with CDS in white and UTR sequences in black. The gRNA site indicated in red appears to be the site targeted by Cas9. (F) Detail of a *lint*^*MI04680*^ terminal cell clone expressing membrane-anchored GFP, which accumulates preferentially at the apical membrane. The arrows indicate discontinuities in the apical membrane. The position of the lethal transgene insertion is indicated in (G). (Scale Bars: A, B 20 μm; A”, B”, C and C’, D-F 5 μm).

We next sought to determine whether loss of *CG8213* could account for the *ich* terminal cell defects. Terminal cell-specific knockdown, using either of two independent RNAi lines, induced numerous apical membrane discontinuities along each branch (Fig 8B–8B”), as well as seamless tube cysts (arrowheads. Fig 8B”). In light of these findings, we rename *CG8213 lumens interrupted* (*lint*). In addition, ~21% of terminal cells (n = 33) exhibited a dramatic decrease in branch number, with the remaining terminal branches containing only isolated inclusions of apical membrane (Fig 8C and 8C’), suggesting a defect in the addition of apical membrane in these terminal branches—similar to the most severe lumen defect observed in *ich* terminal cells (S1B and S1B’ Fig). We confirmed the specificity of these RNAi phenotypes by targeting the *lint* locus region using the CRISPR/Cas9 system (Fig 8D) [89]. Though we did not isolate alleles that deleted the entire coding region by targeting both gRNA sites, we isolated a *lint* mutant allele (*lint*^*Δ4*.*64*^) that behaves genetically as a loss-of-function (see Materials and Methods). We found that *lint*^*Δ4*.*64*^ terminal cells clones exhibited discontinuous lumens (Fig 8D, 8E and 8G) similar to those observed in RNAi experiments, in clones homozygous for a non-complementing, recessive lethal transgene insertion at *lint* (Fig 8F and 8G), and in *ich* clones. We propose that Lint acts downstream of Ich to regulate seamless tube growth and/or maintenance.

To further test if *lint* is the essential target of Ich regulation in tracheal morphogenesis, we asked whether expression of a *lint* cDNA could bypass a tracheal requirement for *ich*. Overexpressing *lint* in otherwise wild-type terminal cells caused liquid-clearance defects (S8A and S8A’ Fig), but did not impact terminal branching or lumen morphogenesis (S8B and S8B’ Fig) with the exception of a few small tube dilations observed in some, but not all, cells (arrowheads in S8B’ Fig). Expression of *lint* in *ich*^*206*^ terminal cells restored seamless tube integrity and shape at a low very low frequency (2% of clones, n = 103, *P =* 0.5, one-sided Fisher’s exact probability test; S8E Fig). The limited rescue observed was expression level-dependent, as no rescue was observed when larvae were raised at 25°C (n = 53 clones; S8E Fig), but the elevated transgene expression observed at 29 ^o^C led to 2% rescue (n = 103 clones, S8D and S8E Fig). Based on these results, we conclude that Ich regulates terminal cell aECM assembly, at least in part, by promoting (directly or indirectly) the expression Lint, a secreted protease that may act in the processing of lumenal matrix proteins.

## Discussion

While extracellular matrices coating the basal aspects of epithelia have long been known to play key roles in organ development and morphogenesis, any potential role of extracellular matrices lining the apical or lumenal aspects of epithelia (aECM) have, by comparison, been largely neglected. Critical gaps in knowledge include: what the components of the aECM are, how they interact with each other and with the apical membrane during assembly, how they are modified over time, and how the aECM regulates morphogenesis and is altered under varying physiological conditions or in disease. In this study we identify an essential role for the aECM in maintaining the shape and integrity of seamless tubes (S9 Fig). Tracheal terminal cells lacking the transcriptional activator Ichor, or its downstream target, Lumens Interrupted, exhibited discontinuous seamless tube fragments, in which the segments of tube that remained intact showed pronounced cystic dilations. TEM analysis demonstrates that aECM organization is compromised in *ichor* deficient larva. Further supporting the hypothesis that it is the aECM factors downstream of Ichor that are relevant to seamless tube morphogenesis, we show that the elimination of chitin, the most abundant aECM component in insects, is sufficient to compromise seamless tube shape and, to a lesser degree, integrity.

Our findings further strengthen the conclusion, prompted by previous studies in nematodes and flies, that the aECM is important for maintaining epithelial integrity. In *C*. *elegans* embryos, the so-called sheath at the apical surface of the epidermis maintains epidermal integrity during cell shape changes that elongate embryos along their anteroposterior axis [90–93]. In the *Drosophila* trachea [54] and *C*. *elegans* excretory system [91], lumenal matrices form scaffolds that maintain the integrity of intercellular contacts joining unicellular tubes.

Do lumenal matrices regulate seamless tube integrity in vertebrates? Endothelial cells secrete a lumenal matrix known as the glycocalyx, which has been implicated in lumen expansion in multicellular blood vessels [40]. In endothelial tip cells, blood flow is required to expand seamless tubes [9,10]. Interestingly, during this initial lumen growth, seamless tube stability is quite labile, undergoing temporary collapse during changes in blood pressure [9, 10]. The endothelial glycocalyx, which promotes lumen expansion through electrostatic repulsion of lumenal surfaces [40], is still maturing at the onset of blood flow [94]. It is possible that an immature glycocalyx contributes to seamless tube collapse during vessel anastomosis. Alternatively, the glycocalyx, a known mediator of shear stress response in endothelial cells [95], could help transduce hemodynamic mechanical signals needed to promote the inward blebbing of lumenal membrane in endothelial tip cells [10]. It would be interesting to determine whether an intact glycocalyx is a prerequisite for stable lumen expansion in endothelial tip cells.

The mechanisms by which the aECM promotes tube integrity in seamless tubes are not known. We favor two non-exclusive possibilities: first, we propose that the aECM may serve as a scaffold capable of dissipating tension along the apical membrane, and second, we propose that the lumenal matrix may play a more direct role in regulating the growth of seamless tubes. In *Drosophila* terminal cells that ramify over areas of 100s of square microns, seamless tubes are likely under considerable tensile stress. These tensile forces are far from static, changing in both magnitude and direction during tube growth as well as during larval locomotion. Against such dynamic tensile strain, tube integrity must be maintained for proper gas-exchange. In larger tube types (autocellular and multicellular), cell junctions are thought to serve as a critical counter force resisting tensile stress; however, along the length of seamless tubes there are no cell junctions. The aECM, with its physical connections to the apical membrane, is ideally positioned to distribute the forces along the length of the tube and in that manner perhaps avoid local forces strong enough to fragment the tube.

We also think it likely that the aECM plays a more informative role in regulating tube growth. In gain-of-function experiments, Ich overexpression arrested the growth of seamless tubes in terminal cells without perturbing lumen continuity. These data suggest that seamless tube growth in terminal cells is sensitive to the levels of Ich effectors, possibly including lumenal matrix factors. A model in which a lumenal matrix merely maintains expanded terminal cell lumens cannot readily account for defects in apical membrane biogenesis, suggesting that components of the aECM may help to organize a membrane platform promoting the growth of the apical domain in terminal cells. Indeed, in loss of function studies, the most severely affected *ich* or *lint* terminal cells showed scattered inclusions of apical membrane, consistent with a failure in the addition of new apical membrane to the growing blind end of terminal cell seamless tubes. This would be consistent with previous work demonstrating that the aECM and the cortical actin meshwork underlying the apical membrane are in communication [58, 96, 97].

The question of how aECM change and mature over time is also one that applies broadly across tubular organs and phyla. For example, in order for the mammalian lungs or the *Drosophila* tracheal system to become functional, their lumens must transition from fluid-filled to gas-filled. Conserved mechanisms mediate this transition, such as liquid and salt reabsorption via epithelial Na^+^ channels [98–102]. The transition from fluid-filled to gas-filled lumens also entails secretion and modification of a lumenal matrix. The *ichor* mutant has a specific defect in tracheal tube maturation without affecting earlier tracheal morphogenesis. Others [64, 75, 103] have reported that mature aECM assembly is required for tracheal liquid clearance and/or gas-filling. In our mosaic analysis, we found *ich* to be required only in larval terminal cells but to be dispensable for tracheal specification, branching, and lumen morphogenesis during embryogenesis. On the other hand, pan-tracheal knockdown of *ich* blocked tracheal gas-filling and resulted in one or more breaks in the dorsal trunks of first instar lavae, suggesting a tissue autonomous but non-cell autonomous role of Ich in larger tubes (presumably reflecting lumenal secretion of Ich target genes). Thus *ich* is broadly required during the maturation of the trachea at the end of embryogenesis or shortly after hatching. This phenocritical phase for *ich* coincides with tracheal aECM maturation (cuticle deposition) and fits with our EM data demonstrating that aECM is disorganized in the trachea of larvae deficient for *ich*. The mature tracheal aECM is thought promote the liquid-to-gas transition by promoting *de novo* gas bubble generation via cavitation [103]. By this process, a column of lumenal liquid must be ruptured at some point to generate a gas-bubble, requiring a decrease in the pressure gradient required to rupture the liquid column at the liquid-cell interface (also called tensile strength). Both the hydrophobic envelope layer of the cuticle, as well as its convoluted taenidial folds, at the surface-liquid interface are predicted to decrease the tensile strength of the lumenal fluid [103]. Tracheal gas-filling takes place with temporal and spatial stereotypy [17], implying the execution of an equally stereotyped developmental program. We propose that Ichor helps ensure this timely transition by initiating the transcriptional activation of a suite of genes promoting mature aECM assembly.

Maintaining gas-filled respiratory lumens in insects and mammals is most critical in the finest lumenal spaces, such as lung alveoli and tracheal terminal cells. In the mammalian lungs, the fine lumens of alveoli impose high surface tension at the air-liquid interface, threatening collapse of these air-filled lumens. To prevent the collapse of these fine lumens, specialized pneumocytes secrete a matrix of phospholipids and proteins that form a lumenal surfactant matrix [104]. Failure to clear lumenal lung liquid or secrete the surfactant matrix underlies lethal respiratory distress syndromes in neonates. The fine lumens of terminal cells exhibit variable regions of fluid-filling, especially at the tips of terminal branches where the lumens are finest [67,105]. Analogous to the alveolar spaces of mammalian lungs, the fine lumenal spaces of *Drosophila* terminal branches may impose high surface tension in a surface-associated aqueous phase, causing the collapse of gas-filling in limited portions of terminal branches. An Ich-dependent lumenal matrix may be required to limit the expansion of these fluid-filled regions throughout the terminal cell, by reducing the surface tension in a lumenal-surface-associated aqueous phase.

Modifications to a lumenal matrix are a conserved process underlying the functional maturation of tubular organs. In the *Drosophila* trachea [57–59, 81] and *C*. *elegans* excretory system [41, 91, 106, 107] transient aECMs give way to stable ones at the completion of embryogenesis. However, the developmental signals controlling such matrix reorganizations remain poorly understood. In the trachea, this process presumably entails the upregulation of enzymatic processes, such as proteolysis and chitin hydrolysis, at the end of embryogenesis to clear the chitin filament. Endocytosis is then required to clear material from tracheal lumens [17]. We propose that Ichor and its as-yet-unknown cooperating transcription factors (see above) help initiate stable aECM assembly in the trachea by transcriptionally activating a suite of genes, such as Lint, involved in cuticle biogenesis.

While zinc finger transcription factors are abundant throughout the animal kingdom, Ich does not have clear orthologs beyond insects; however, the aECM targets whose expression Ich regulates may be more closely conserved. The domain organization of Lint, for example, is similar to that observed in a family of vertebrate type II transmembrane serine proteases (TTSPs). Vertebrate TTSPs are defined structurally by an N-terminal single-pass transmembrane domain separating a short cytosolic domain from a larger extracellular domain containing a trypsin protease domain at its C-terminus [108]. Genome sequencing in mouse and humans has identified ~20 TTSPs in mouse and humans. Lint shows 32–37% identity in its C-terminal trypsin protease domain to multiple mouse and human TTSPs, including TMPRSS11f, ST14 (Matriptase), TMPRSS9, and mouse TMPRSS11C. Lint differs from canonical TTSPs in that it lacks the variable array of protein-protein interaction domains found in their extracellular domains. But, like Lint, vertebrate TTSPs such as Matriptase are expressed broadly in epithelia, suggesting a conserved role for secreted transmembrane proteases in the development or physiology of epithelial tissues [108]. Indeed, membrane-anchored proteases have a conserved role in the assembly and maintenance of epithelial barriers. Lint is required for cuticle assembly in the pupal wing, where it is proposed to play an in instructive role, either directly or indirectly, in organizing the multi-layered aECM [73]. Similarly, in mice, TTSPs such as Matriptase [109, 110] and TMPRSS11f [111] are essential for the formation and/or function of an extracellular barrier in the mammalian cornified envelope. The cornified envelope is a complex meshwork of terminally differentiated non-living keratinocytes, called corneocytes, that are chemically cross-linked with a heterogeneous array of structural proteins and lipids. Together, these structures form a water-impermeable barrier preventing unregulated inside-out and outside-in passage. The cornified envelope serves the same physiological function as the extensively chemically cross-linked and hydrophobic insect cuticle. The murine Matriptase homolog is thought to play a role in the secretion of extracellular matrix material needed to maintain epidermal barrier integrity [110]. Our work implicates Lint-mediated lumenal matrix assembly in the integrity of seamless tubes. Interestingly, Matriptase has been implicated in neural tube closure and angiogenesis [112], suggesting a possible conserved role for membrane-anchored serine proteases in tubulogenesis.

## Materials and methods

### Fly stocks

To generate GFP-labeled mitotic clones in the tracheal system, the following stocks were used: *ywFLP*^*122*^*; btl>GFP; FRT*^*82B*^ *tubGAL80* and *ywFLP*^*122*^*; btl>GFP*, *Wkd-mKate2; FRT*^*82B*^ *P{tubGAL80}* and *ywFLP*^*122*^*; btl>moeABD-GFP; FRT*^*82B*^ *P{tubGAL80}* and *ywFLP*^*122*^*; FRT*^*G13*^ *P{tubGAL80}; btl>CD8-GFP* to generate MARCM clones (Lee and Luo, 2001) or *ywFLP*^*122*^*; btl>GFP*,*DsRednls;FRT*^*82B*^ *UAS-GFPi* (Ghabrial et al, 2011); *btl>GFP; FRT*^*2A*^ *FRT*^*82B*^ and *btl>GFP; FRT*^*82B*^ *kkv*^*sob*^ stocks are described in [67]. An *FRT*^*82B*^ *ich*^*206*^ *sr e ca/TM3* stock was generated by recombining an *FRT*^*82B*^ *ich*^*206*^ chromosome [67] with an isogenic *FRT*^*82B*^ *cu sr e ca* chromosome. *FRT*^*82B*^ *ich*^*543*^/*TM2* was recovered from a non-complementation screen (see below). *UAS-ChtVisT-TdTomato* was a generous gift from Paul Adler’s lab [68]. *w; Df(3R)osk* was a generous gift from Paul MacDonald’s lab (U. Texas at Austin) [70] and was recombined onto the *FRT*^*2A*^ *FRT*^*82B*^ chromosome. The *P{PZ}l(3)05652* insertion was provided by the Bloomington Stock Center (Bloomington, IN, USA) and was recombined onto the *FRT*^*2A*^ *FRT*^*82B*^ chromosome. For RNA interference experiments, the following strains [113] were obtained through the Bloomington Stock center: *y*^*1*^ sc* v^*1*^*; P{y[+t7*.*7] v[+t1*.*8] = TRiP*.*HMS02762}attP2* carrying a dsRNA against *ich* under UAS control; *y*^*1*^ sc* v^*1*^*; P{y[+t7*.*7] v[+t1*.*8] = TRiP*.*HMJ22360}attP40* and *y*^*1*^ sc* v^*1*^*; P{y[+t7*.*7] v[+t1*.*8] = TRiP*.*HMC04037}attP40*, expressing independent dsRNAs against *lint*. *ru*^*1*^ *th*^*1*^ *st*^*1*^ *Df(3R)3-4/TM3* was provided by the Bloomington Stock center. A recessive lethal transgene insertion at *lint*, MI04680, was obtained from Bloomington Stock Center and recombined onto a *FRT*^*40A*^*FRT*^*G13*^ chromosome provided by Bloomington Stock Center. The following GAL4 drivers were used: *btl-GAL4* [71], *SRF-GAL4* (a gift from Mark Metzstein, U. Utah, Salt Lake City, UT, USA), and *drumstick-GAL4* [114] (Bloomington Stock Center). For making germline clones of *ich*, the w*; FRT^*82B*^ *P{ovoD1-18}/st*^*1*^ *βtub85D*^*D*^ *ss*^*1*^ *e*^*s*^*/TM3* [115] was obtained from Bloomington Stock Center. An isogenic *w*^*1118*^ wild-type strain was obtained from Bloomington Stock Center.

### Mosaic analysis

Positively marked clones were generated in the tracheal system as previously described (Ghabrial et al, 2011). Briefly, embryos were collected for 4h at 25C, then heat-shocked 1h at 38°C to induce FLPase expression. Embryos were aged at 25C until the wandering third instar unless otherwise indicated. Mosaics larvae were identified using a Leica MZ16F fluorescence stereomicroscope. Germline clones were generated using the dominant female-sterile technique [115]: briefly, *hsp-FLP*^*122*^*; FRT*^*82B*^ *P{ovoD*^*1-18*^*}/FRT*^*82B*^ *ich*^*206*^ *sr e ca* second and third instar larvae were heat-shocked twice for 2h at 38°C, over the course of two days. Females with mosaic germlines were crossed to *FRT*^*82B*^ *ich*^*206*^/*TM3 twi>GFP* males, embryos were collected and maternal/zygotic null animals identified by lack of GFP expression.

### Allele screen

To facilitate mapping of the *ich* locus, ethylmethanesulfonate (EMS)-induced mutations were screened for non-complementation of the original *ich*^*206*^ recessive lethal allele [67]. ~50 Males from an isogenic *btl>GFP; FRT*^*2A*^ *FRT*^*82B*^ strain were fed 25 mM EMS in a sucrose solution overnight. These males were then mated to ~100 *btl>GFP; Pr hs-Hid/TM3 P{tubGAL80}* females [67]. Eggs were collected over the course of several days, then heat-shocked to kill any *hs-Hid* animals. Approximately ~1000 mutagenized *FRT*^*2A*^ *FRT*^*82B*^ chromosomes were then screened for the presence of a recessive lethal mutation(s) that failed to complement the tester chromosome carrying the *ich*^*206*^ allele. One additional allele, *ich*^*543*^, was recovered. This allele was also for complementation with *Df(3R)osk*, which uncovers *ich*, and with *P{PZ}l(3)05652*, a hypomorphic P-element induced allele of ich. The *ich*^*543*^ allele failed to complement all of these independently derived *ich*-deficient chromosomes.

### Mapping and genetic characterization

#### Mapping studies

Meiotic recombination was used to narrow down the candidate gene interval on chromosome 3R. *FRT*^*82B*^ *ich*^*206*^*/TM3* flies were crossed to an isogenic *FRT*^*82B*^ *cu*^*1*^ *sr*^*1*^ *e*^*s*^ *ca*^*1*^ strain (Bloomington Stock Center) to generate *FRT*^*82B*^ *ich*^*206*^*/FRT*^*82B*^ *cu*^*1*^ *sr*^*1*^ *e*^*s*^ *ca*^*1*^ females, which were then crossed to *ru*^*1*^ *h*^*1*^ *th*^*1*^ *st*^*1*^ *cu*^*1*^ *sr*^*1*^ *e*^*s*^ *Pri*^*1*^ *ca*^*1*^*/TM6B* (Bloomington Stock Center) males to recover recombinant chromosomes. Recombinant chromosomes were scored for recessive visible markers and then tested by complementation against the original *ich*^*206*^ mutant chromosome. The map was further refined using chromosomal deletions, and a recessive lethal lesion at 85B7-85B8 was identified. Genomic DNA was isolated from *ich*^*206*^ mutant embryos (Qiagen DNeasy Blood and Tissue Kit) and subjected to whole-genome sequencing (Otogenetics, Atlanta, GA). A non-synonymous mutation in the coding region of CG11966 at cytological position 85B8-85B9 was found. To confirm gene identity, a lethal insertion at CG11966, *P{PZ} l(3)05652*, was tested and found not to complement *ich* lethality. Finally, genomic DNA from *ich*^*543*^ homozygous embryos was isolated (Qiagen DNeasy Blood and Tissue Kit) and the genomic region encompassing CG11966 was PCR amplified and sequenced. See S1 Table for a list of primers used. All PCR reactions were carried out using either Taq DNA Polyerase (Sigma-Millipore) or Platinum Taq DNA polymerase (Invitrogen). Prior to sequencing, excess primers and nucleotides were removed from the reaction products using Exo-SAP-IT (Thermofisher Scientific), or desired amplicons were gel-extracted using glassmilk (MP Biomedical, Santa Ana, CA, USA). PCR amplicons were sequenced using ABI sequencers (Applied Biosystems). Sequencing data were analyzed using Sequencher software.

#### Allele characterization

To characterize *ich* allele strengths, hatching rates were determined in various *ich* mutant allele combinations. Eggs were collected for 16h at 25C and the embryos were allowed to develop for another 24h at 25°C. Hatched first instar larvae were then genotyped by GFP expressed from a TM3 *twi>GFP* balancer chromosome. Tracheal phenotypes were visualized by brightfield in whole-mount larval preparations using a Leica DM5500 B upright widefield epifluorescence microscope (Leica Mirosystems). Both *ich*^*206*^ and *ich*^*543*^ are embryonic lethal in homozygotes and hemizygotes in *trans* to *Df(3R)osk*. The *ich*^*206*^*/ich*^*543*^ transheterozygotes showed a slight increase in hatch rate but died during the first instar. Thus, both *ich*^*206*^ and *ich*^*543*^ alleles behave as strongly hypomorphic or amorphic alleles. By contrast, viable *P{PZ}l(3)05652* homozygous larvae with gas-filled trachea hatch at the expected Mendelian frequency, while *P{PZ}l(3)05652* hemizygotes showed increased embryonic and first instar lethality, consistent with *P{PZ}l(3)05652* behaving as a weak hypomorphic allele.

To characterize *kkv*^*sob*^ alleles, genomic DNA from embryos homozygous for each of the *kkv*^*sob*^ alleles was isolated using a Qiagen DNeasy Blood and Tissue kit. The *kkv* coding region was amplified by PCR and sequenced using the primers described in S1 Table. To functionally characterize the putative null alleles *kkv*^*sob404*^ and *kkv*^*sob483*^, the embryonic tracheal phenotypes of each homoallelic combination was compared to the corresponding hemizygous combination *in trans* to the *Df(3R)3-4* chromosomal deletion. Staining of embryos for Gasp [116] expression (mAb2A12; DSHB, Iowa City, IA) was used to score lumen phenotypes. By this assay, *kkv*^*sob404*^ homozygotes and *kkv*^*sob404*^*/Df(3R)3-4* hemizygotes exhibited identical lumenal defects, consistent with *kkv*^*sob404*^ behaving as a strong hypomorphic or null allele. *kkv*^*sob483*^ homozygotes exhibited a distinct phenotype from *kkv*^*sob483*^/*Df(3R)3-4* hemizygotes: namely, the showed reduced lumenal accumulation of Gasp.

### Cuticle preparations

*FRT*^*82B*^ *kkv*^*sob483*^*/TM3 twi>GFP*, *FRT*^*82B*^ *ich*^*206*^*/TM3 twi>GFP*, or *FRT*^*82B*^ *ich*^*543*^*/TM3 twi>GFP* adults were inter-crossed and eggs collected on apple juice agar plates for 6h at 25°C. Eggs were aged further for 16h at 29°C. Hatched first instar heterozygous control larvae were picked and placed in 1:1 methanol: heptane. Mutant embryos were sorted under a fluorescence stereomicroscope by the absence of GFP expression and dechorionated in 50% bleach for 1.5 min. Embryos were devitellinated in 1:1 methanol: heptane and washed 3–4 times in 100% methanol. Methanol was replaced with 0.1% PBS-Tween-20 (PBS-Tw) and embryos/larvae were allowed to settle for 10min at room temperature. Cuticles were expanded in PBS-Tw at 65°C for 20 min. PBS-Tw was replaced by 50 μl Hoyer’s medium. Embryos/larvae were incubated in a 2:1:1 mixture of Hoyer’s mountant: lactic acid: dH_2_O for 16-24h at room temperature and were then mounted on slides. Preparations were visualized by phase contrast using an Evos FL Auto Imaging microscope.

### Transgenic constructs

The entire coding sequences of CG11966 (RE65372) and CG8213 (LD43328) were obtained as cDNAs from the *Drosophila* Genomics Resource Center (DGRC, Bloomington, IN, USA, NIH grant 2P40OD010949). To generate pUAST-ich, EcoRI and KpnI restriction sites were added during PCR amplification (see S1 Table). The amplicon was directionally subcloned into the pUAST vector (Brand and Perrimon, 1993). To generate pUAST-CG8213, the full-length cDNA insert was subcloned into pUAST at the EcoRI and XhoI restriction sites. To generate Flag-tagged Ich, the EcoRI site, start codon, and Flag epitope were added to the forward primer, and a KpnI site was included in the reverse primer (see S1 Table). The Ich-Flag PCR product was TA cloned (TOPO-TA cloning kit, Invitrogen) and then subloned into pUAST (EcoRI, KpnI). Sanger sequencing was used to verify the final constructs (S1 Table).

Overlap extension PCR [117] was used to generate the Flag-VP16-IchDBD and EnR-IchDBD-Flag fusions (for primers, see S1 Table). The following plasmid templates were used in the PCR strategy: pActPL-VP16AD (Addgene #15305) [78], *en* cDNA clone LD16125 (DGRC), and *ich* cDNA clone RE65372 (DGRC). EcoRI and KpnI restriction sites were added during PCR. PCR amplicons of the expected size were TA cloned (TOPO TA kit, Invitrogen) and subsequently subcloned into pUAST (EcoRI, KpnI). A start codon, flag sequence, and SV40 nuclear targeting signal were added to the VP16 transcriptional activation domain during PCR. An SV40 nuclear targeting sequence, followed by a flag sequence and stop codon were added to IchDBD during PCR for the EnR-IchDBD-Flag fusion.

Transgenic strains for UAS-ich, UAS-Flag-ich, UAS-Flag-VP16-IchDBD, UAS-EnR-IchDBD-Flag, and UAS-CG8213 were generated by P-element transformation (Rainbow Transgenic Services, Camarillo, CA, USA).

### Generation of *lint* mutant allele by CRISPR/Cas9

Optimal gRNA target sites in the *Drosophila melanogaster* genome (release 6) were selected using the default settings of the flyCRISPR Optimal Target Finder web program (http://tools.flycrispr.molbio.wisc.edu/targetFinder/). Genomic DNA from a *w*^*1118*^*; FRT*^*40A*^ *FRT*^*G13*^ stock (Bloomington Stock Center) was isolated using a Qiagen DNeasy Blood and Tissue kit. Genomic DNA flanking candidate gRNA sites was amplified and sequenced to rule out gRNA sites containing single-nucleotide polymorphisms. A site upstream of the transcription start site (GCCATGGACACCAACTGATTCGG) and a site within the 3’ UTR (GCATTTCAAACGACATTCGCCGG) common to all spliceoforms were chosen. 5’ phosphorylated oligonucleotides were synthesized by Integrated DNA technologies (Coralville, IA, USA) according to [89]. 5’ gRNA plasmids were designed according to [89]. Single colonies of pU6-BbsI-gRNA [89] transformants were selected for plasmid isolation (GenElute Minipreparations, Sigma Millipore) and validated by sequencing. A *y*^*1*^ *M{nos-Cas9}; FRT*^*40A*^*FRT*^*G13*^ stock was constructed using the *y*^*1*^ *M{nos-Cas9}* stock [118] (Bloomington Stock Center) expressing the Cas9 nuclease from a germline promoter. *y*^*1*^ *M{nos-Cas9}; FRT*^*40A*^*FRT*^*G13*^ embryos were injected with a mixture of two gRNA plasmids by Best Gene, Inc. (Chino Hills, CA, USA). Single GO’s were outcrossed to *al*^*1*^ *dp*^*ov1*^ *b*^*1*^ *p*^*1*^ *Bl*^*1*^ *c*^*1*^ *px*^*1*^ *sp*^*1*^*/SM1* (Bloomington Stock Center) flies and single G1’s were back-crossed to establish individual lines, which were tested by genetic complementation against multiple deficiencies uncovering CG8213, including *Df(2R)Np5 In(2LR) w*^*45-32n*^ and *Df(2R)H3E1* (Bloomington Stock Center), as well as a recessive lethal Minos insertion at CG8213 (*y*^*1*^*w*^*1*^*; CG8213*^*MI04680*^). PCR (see S1 Table for primers) analysis suggested that the 5’ gRNA site was targeted, while the 3’ gRNA site was left intact. However, the exact nature of the lesion remains unclear due to difficulties in PCR amplifying gDNA flanking the 5’ gRNA site from mutants.

### Antibodies and immunofluorescence

#### Rat α-Trh

To generate the α-Trh polyclonal sera, the peptide C- SFHLYHKGSPASGWYSTPS was selected and used to immunize 3 rats, which were boosted and exsanguinated (Bio-Synthesis). The sera from primary bleeds were diluted at 1:10 in PBS and pre-adsorbed against wild type embryos.

#### Larval fillets for antibody staining

Third instar larvae were filleted in cold 1X PBS on Sylgard-coated dishes using dissection pins (Minuten, Ento Sphinx). Briefly, larvae were pinned down ventral side up. A longitudinal incision using fine scissors (Fine Science Tools, Foster, CA, USA) was made along ventral epidermis and the resulting epidermal pelt was pinned down on four sides. Forceps were used to clear away extraneous tissue, leaving the tracheal tree. Larvae were fixed in the dish in 4% paraformaldehyde (Electron Microscopy Sciences, Hatfield, PA, USA) in 1X PBS for 15 min on ice. Fillets were unpinned and permeabilized in 0.3% Tween-20/0.3% Triton-X-100 in 1XPBS (PBST) for 15 min at room temperature. Fillets were then incubated overnight at 4C in primary antibody diluted in PBST. Following washes in fresh PBST (4X 15 min or 3X 30 min), secondary antibody incubations were done for 1.5-2h at room temperature or overnight at 4C, followed by washes in PBST at room temperature. Fillets were mounted in Aqua Polymount medium (Polysciences, Inc, Warrington, PA).

#### Embryos for antibody staining

Egg lays were collected at 25°C on apple juice plates and aged to enrich for embryos of the appropriate stages. Eggs were then dechorionated in 50% bleach for 1.5–3 min, followed by formaldehyde fixation for 25 min. Embryos were then devitellinated in 1:1 heptane:methanol mixture. Embryos were successively rehydrated using 5 min washes in 80% methanol: 20% 1XPBS, 60% methanol: 40% 1X PBS; 40% methanol: 60% 1X PBS, 20% methanol: 80% PBS-Tween 20 (0.05%), 0.05% PBS-Tween-20. Embryos were blocked in 4% horse serum in 0.05% PBS-Tw for 1h at room temperature or overnight at 4C, then incubated overnight at 4C in primary antibody. Following washes in fresh 0.05% PBS-Tw (3X 30 min), secondary antibody incubations were performed for 2-3h at room temperature.

#### Antibodies and working dilutions

The following primary antibodies were used in this study: Chicken anti-GFP IgY (1:1000, Life Technologies A10262, Carlsbad, CA, USA); Rabbit anti-Whacked peptide (1:750) [69]; rabbit anti- aPKC ζ H-300 (1:200, Santa Cruz); mouse anti-aPKC ζ A-3 (1:200, Santa Cruz); mouse anti flag M2 (1:1000, Sigma Aldrich); mouse anti- Acetylated tubulin clone 6–11 B-1(1:2000, Sigma Aldrich); Rabbit anti-Verm (1:500) [49]; mAb2A12 (1:5, DSHB, Iowa City, IA); rat anti-Trh (1:500 final dilution, this study**)**; mouse anti-β-galactosidase (1:5000–1:1000; Millipore-Sigma**).** The following secondary antibodies (Life technologies) were used: goat anti-chicken Alexa 488, donkey anti-mouse IgG Alexa 555, goat anti-rat Alexa 555, donkey anti-rabbit Alexa 647, and donkey anti-mouse IgG Alexa 647 (1:1000 each). To visualize chitin in fixed embryos, a TMR Star-conjugated chitin-binding probe (NEB) was incubated along with secondary antibodies (1:1000).

### Microscopy

Mosaic larvae were identified by direct fluorescence using a Leica MZ16F fluorescence stereomicroscope (Leica Microsystems). To visualize terminal cells in wholemount specimens, third instar larvae were placed in a drop of 60% glycerol in 1X PBS, then heat-killed at 70C for ~12s, and flattened under a coverslip. Larvae were then imaged using direct fluorescence and Brightfield optics using a Leica DM5500 B upright or A Leica DM6000 inverted widefield epifluorescence microscope (Leica Mirosystems). Images were acquired using either a Leica DFC360FX camera or a Hammamatsu Orca-R2 Digital CCD camera (C10600, Hamamatsu Photonics). Z-stacks were captured and processed by deconvolution using Leica Advanced Fluorescence Application Suite (Leica Microsystems). For most images, a single deconvolved z-slice is shown, except where projected z-stacks were used to capture whole-cell detail.

To analyze terminal cell lumen ultrastructure by TEM, *SRF>eGFP*,*ich* RNAi first instar larvae were subjected to a high pressure freezing/freeze substitution protocol as described in [63]. *SRF>eGFP* and *btl>GFP* larvae were processed as a representative wild-type controls. 45–70 nm-thick sections were imaged at 125 keV using a Hitachi 7200 electron microscope.

### RNA *in situ* hybridizations

RNA *in situ* hybridizations were performed on St.13-16 embryos according to the protocol described by [119]. Briefly, DIG-labeled RNA probes were synthesized from PCR templates amplified from cDNA clones for full-length *ect* (RE01075), CG8213 (LD43328), *osi18* (RE07882), and *osi19* (RE01054) obtained from the DGRC. Primers including T3 and T7 promoters were designed according to [120]. See S1 Table for description of PCR primers. PCR products were resolved by agarose gel electrophoresis and purified using glassmilk (MP Biomedicals). *in vitro* transcription reactions were performed using T3 (anti-sense) and T7 (sense) DNA-dependent RNA polymerases (Promega corp., Madison, WI). RNA was labeled with Digoxigenin (Roche Applied Science, Indianapolis, IN). Quality of *in vitro* transcription products was assessed by agarose electrophoresis and the RNA precipitated using ethanol.

Eggs were collected on apple juice plates for 6-7h at 25C, then aged for ~16h at 18°C. Embryos were manually sorted under a fluorescence stereomicroscope to assess genotype (*TM3 twi>GFP* balancer). Embryos were dechorionated in 50% bleach for 1.5 min and fixed for 25 min in a formaldehyde and heptane mixture. Embryos were devitellinated using a 1:1 heptane:methanol mixture. Embryos were then processed for probe hybridization according to [119]. ~50 ng of DIG-labeled RNA probe was diluted in 100 μl hybridization buffer. RNA probe signal was detected by an Alkaline Phophatase (AP) reaction using nitroblue tetrazolium (Roche Applied Science) and bromochloro indoyl phosphate (Roche Applied Science) in 0.1M Tris-Cl, 0.1 M NaCl, 0.05M MgCl_2_ in 0.1% PBS-Tween-20, pH 9.5. For CG8213, *ect*, and *osi19* probes, the AP reaction was developed for 4.5h at room temperature, while for *osi18* probes, the AP reaction was developed for ~16h at room temperature. Embryos were mounted in 60% glycerol in 1X PBS and imaged using Brightfield microscopy.

### Statistical analysis

For cDNA rescue experiments, the significance of categorical frequency data was determined using Fisher’s exact probability tests (http://vassarstats.net). one-sided P values were reported under the assumption that rescue conditions will deviate from mutant conditions in one direction. For frequency data with more than 2 possible outcomes, Chi-Square tests were used.

## Supporting information

S1 Fig *ichor* terminal cells exhibit multiple tube defects. (A-A’) *ich*^*206*^ terminal cell clone exhibiting severe pruning (A) and tube growth defect (A’). (B,C) Wild-type control (C) and *ich*^*206*^/Df(3R)*osk* (D) embryonic terminal branches visualized by fluorophore-conjugated chitin-binding probe. *ich* terminal cells are able to extend seamless tubes (arrowheads) beyond the cell body, as marked by the terminal cell nucleus (Trh). Larval (D-D”) *ich*^*543*^ terminal cell clone exhibiting an accumulation of multiple lumens, often with convoluted trajectories (arrowheads in D’, D”). (E-G’) GFP-labeled *ich*^*206*^ MARCM tracheal clones visualized in wholemount heat-killed larvae. Fluorescent images are superimposed on brightfield. Loss of ich in isolated dorsal trunk (E, E’), autocellular (F, F’), and fusion branch (G, G’) cells caused no gas-filling or overt lumen defects. (H-I’) Wholemount heat-killed *btl>DsRed* (H,H’) and *btl>DsRed*, *ich* RNAi (I,I’) first instar larvae (L1). In contrast to wild-type controls (H,H’*)*, *btl>DsRed*, *ich* RNAi L1s exhibit pan-tracheal liquid clearance defect (I) as well as a breaks in the dorsal trunks (outlined in I’). (J) Comparison of lethal phase for ich loss-of-function alleles. (Scale Bars: A,D 20 μm; B,C 10 μm; A’,D’, D” 5μm; E-G’, 50 μm).
(TIF)

S2 Fig EnR-IchDBD-FLAG, but not full-length Ich, induced apical membrane discontinuities. (A-B’) *btl*>GFP,EnR-IchDBD; 2*FRT* terminal cell clones stained for GFP, the apical membrane using Wkd antiserum (A’), and the Flag epitope (B,B’). *btl*>GFP, EnR-IchDBD; 2*FRT* terminal cell clones exhibit cystic, discontinuous lumens (A,A’). EnR-IchDBD-FLAG localizes to the nucleus of terminal cells (B, B’). (C-D’) Control *btl*>*GFP*, *Wkd-mKate2*; 2*FRT* (C,C’) and *btl*>*GFP*, *Wkd-mKate2*, *ich*; 2*FRT* (D,D’) terminal cell clones overexpressing Ich. In contrast to wild-type controls (C,C’), terminal cells overexpressing full-length Ich (D,D’) exhibit severe pruning with rudimentary lumens (D’). (E-E”‘) *btl*>*moe-GFP*, *ich*; 2*FRT* terminal cell clones stained to label cortical f-actin (E’), cortical acetylated tubulin (E”), and aPKC (E”‘). Unlike EnR-IchDBD, full-length Ich overexpression in terminal cells does not perturb lumen patency but does disrupt localization of certain apical membrane markers, such as aPKC. (Scale Bar: A-B’, E-E”‘ 5μm; C-D’, 50 μm).
(TIF)

S3 Fig An *ich*::*nLacZ* transcriptional reporter is expressed in cuticle-secreting epithelia. Embryos heterozygous for the *ich*::*nLacZ* P element enhancer trap insertion were immunostained for nuclear LacZ (nLacZ, green) and the tracheal specific transcripton factor Trachealess (Trh, red). (A,A’) LacZ signal is first detected in Stage 10 embryos in broad epidermal stripes (A’). During germband retraction (B, B’), epidermal expression is strongest in the T2, T3, and A8 epidermal parasegments (arrowheads in A-B’). LacZ reporter expression is not detected during primary branching (B-C’). Pan-tracheal LacZ expression is first detected at St. 14 (D, D’) and continues during later stages (St. 15: E, E’), coinciding with lumen growth and cuticle deposition. In addition to tracheal expression, LacZ is also expressed in the epidermis (arrowhead in E’), foregut (F, G), and hindgut (arrowhead in H). All are ectodermally-derived epithelia that secrete chitin-based cuticles. (Scale Bars: 20 μm).
(TIF)

S4 Fig *ichor* is dispensable for the formation and modification of the tracheal chitin cable. (A-B’) Wild type (WT) (A,A’) and *ich*^*206*^/Df(3R)*osk* (B,B’) embryos immunostained for Trachealess (white) and chitin-binding probe (CBP, red). *ich*^*206*^/Df(3R)*osk* embryos deposit a wild-type chitin filament and exhibit neither cystic nor convoluted lumens. (C, C’) *kkv*^*sob483*^/Df(3R)3-4 embryos stained for chitin-binding probe (red) and lumenal matrix protein Gasp (mAb2A12), showing cystic lumen morphology characteristic of chitin-deficient embryos. (D, E) Control (D) and *ich*^*206*^ embryos stained for Gasp, showing *ich* is dispensable for lumenal accumulation of Gasp. (F-F’) *ich*^*206*^/Df(3R)*osk* hemizygotes stained for Trh and DE-cadherin (red) and the Chitin Deacetylase Verm (magenta). *ich* is dispensable for luminal accumulation of Verm. (G,J) Maternal-zygotic *ich*^*206*^ mutant embryos (H) exhibit wild-type lumen morphogenesis in the embryonic trachea. Restoring zygotic *ich* expression in maternally-deficient embryos (G) has no effect on tracheal lumen morphogenesis. (Scale Bars: 10 μm).
(TIF)

S5 Fig Characterization of *kkv*^*sob*^ alleles. (A-C’) GFP-labeled *kkv*^*sob404*^ MARCM clones in wholemount heat-killed third instar larvae. Unlike wild-type control terminal cells (asterisk in A’), *kkv*^*sob404*^ terminal cell clones exhibit a cell-autonomous gas-filling defect (arrow in A’). Isolated *kkv*^*sob404*^ clones in the dorsal trunk (B, B’) causes cell-autonomous ‘divots’ (arrows in B’) in the gas-filled lumen. Cell-autonomous loss of *kkv* in autocellular branches (C,C’) causes a cell-autonomous gas-filling defect (arrow in C’). (D-G’) *btl*>*GFP*; *kkv*^*sob*^ mutant embryos and heterozygous control siblings stained for GFP (green) and chitin-binding probe (red). *kkv*^*sob*^ mutants fail to form the transient chitin filament and exhibit cystic lumens in the dorsal trunk. (H-L) Analysis of lumen morphology in wild-type (H), *kkv*^*sob*^ hemizygous mutants (I and K), and homoallelic mutants (J and L) using mAb2A12. The cystic dorsal trunks of *kkv*^*sob404*^/Df(3R)3-4 hemizygotes (I) resembles that of *kkv*^*sob404*^ homoallelic mutants (J). However, *kkv*^*sob483*^ homozygotes (L) can exhibit a severe reduction of luminal 2A12 staining not observed in *kkv*^*sob483*^ hemizygotes (K). (Scale Bars: A-C’ 50 μm; D-L, 10 μm).
(TIF)

S6 Fig *ich* regulates ectodermal expression in the foregut and epidermis. (A, B, G) Wild-type (WT, *w*^*1118*^) embryos hybridized with DIG-labeled anti-sense (A, B) or sense (G) probe. At Stage 13, *ect* is expressed in the foregut primordium (brackets in A) and posterior spiracles (black arrowhead in A). By Stage 16, *ect* is expressed in all cuticle-secreting epithelia, including the foregut (bracket in B), epidermis (arrow in B), trachea (black arrowhead in B), and hindgut (white arrowhead in B). This signal is specific to *ect* transcript because the corresponding sense probe gives no such pattern (G). (C, D) Control *ich*^*543*^*/+* heterozygotes hybridized with same anti-sense *ect* probe exhibit a wild-type expression pattern. By contrast, (E, F), *ich*^*543*^ homozygotes exhibit reduced *ect* expression in the foregut and epidermis, though *ich* is not absolutely required for tracheal expression (black arrowheads in E, F).
(TIF)

S7 Fig *osi18* and *osi19* expression patterns in wild-type embryos. (A-D) Isogenic wild-type (WT,*w*^*1118*^) embryos hybridized with DIG-labeled *osi18* (A-C) or *osi19* (E-G) antisense probes and corresponding sense probes (D,H). *osi18/19* expression is first detected at approximately Stage14 (A,E) in the dorsal trunk and transverse connectives. At Stages 15 (B, F) and 16 (C,G), *osi18/19* are strongly expressed throughout the tracheal system. This pattern of expression is specific to *osi18/19* transcript because *w*^*1118*^ embryos hybridized with the corresponding sense probes (D, H) give no tracheal signal.
(TIF)

S8 Fig *lint* is one of multiple downstream targets of Ichor in the terminal cell. (A,A’) Wholemount images of heat-killed third instar drm>GFP, lint terminal cells overexpressing lint from a UAS promoter. *lint* overexpression in the terminal cell causes a liquid clearance defect in terminal branches (arrows in A, A’). Unlike terminal cells overexpressing Ich (S2 Fig), *lint* overexpression (B, B’) does not significantly impair terminal branching or lumen growth (B), but does cause dilations of the apical membrane (arrowheads in B’). (C-E) In most instances, restoring expression of *lint* in *ich*^*206*^ terminal cells is not sufficient to suppress tube integrity defects (C, C’). However, elevated transgene expression (D,D’) could restore tube integrity at a non-significant frequency(E, 2% of cells, *P* = 0.5, one-sided Fisher’s exact probability test). (Scale Bars: A, A’ 50 μm; B, C, D 20 μm; B’,C’,D’ 5 μm).
(TIF)

S9 Fig Summary of results. Ichor encodes a zinc finger transcriptional activator (Figs 2 and 4) controlling the assembly of chitin-based cuticles (Figs 3 and S3). In terminal cells, Ichor is dispensable for the secretion of bulk aECM material, but rather, is essential for the organization of this material into an ordered extracellular matrix. In cooperation with other, as yet unidentified transcriptional regulators (compare Fig 6L and 6N), Ichor directly or indirectly activates the expression of known (Fig 7) or putative (Fig 6) regulators of aECM assembly in the trachea. We speculate that Ichor promotes extracellular assembly processes at the lumenal surface of seamless tubes, in part by allowing proteolytic processing events needed for matrix proteins to be incorporated into the terminal cell aECM. The terminal cell aECM is built from a heterogeneous chitinous cuticle organized into a series of taenidial ridges ([63] and Fig 3). When *ich* function is compromised, taenidiae fail to form and seamless tube lumens are occluded with disorganized matrix material (Fig 3). A lumenal matrix is required in terminal cells for the integrity and shape (Figs 1, 5 and 8) of seamless tubes—perhaps by forming a scaffold to dissipate tension acting on seamless tube lumens, as well as possibly forming an organizing scaffold to coordinate cell hollowing.
(TIF)

S1 Table Primers used for sequencing, cloning, and RNA probe production. (XLSX)
