# Supplementary figures and images for: An Ichor-dependent apical extracellular matrix regulates seamless tube shape and integrity
Source: PLoS Genet. 2018 Jan 8;14(1):e1007146. doi: 10.1371/journal.pgen.1007146 (PMC5774827; doi:10.1371/journal.pgen.1007146)

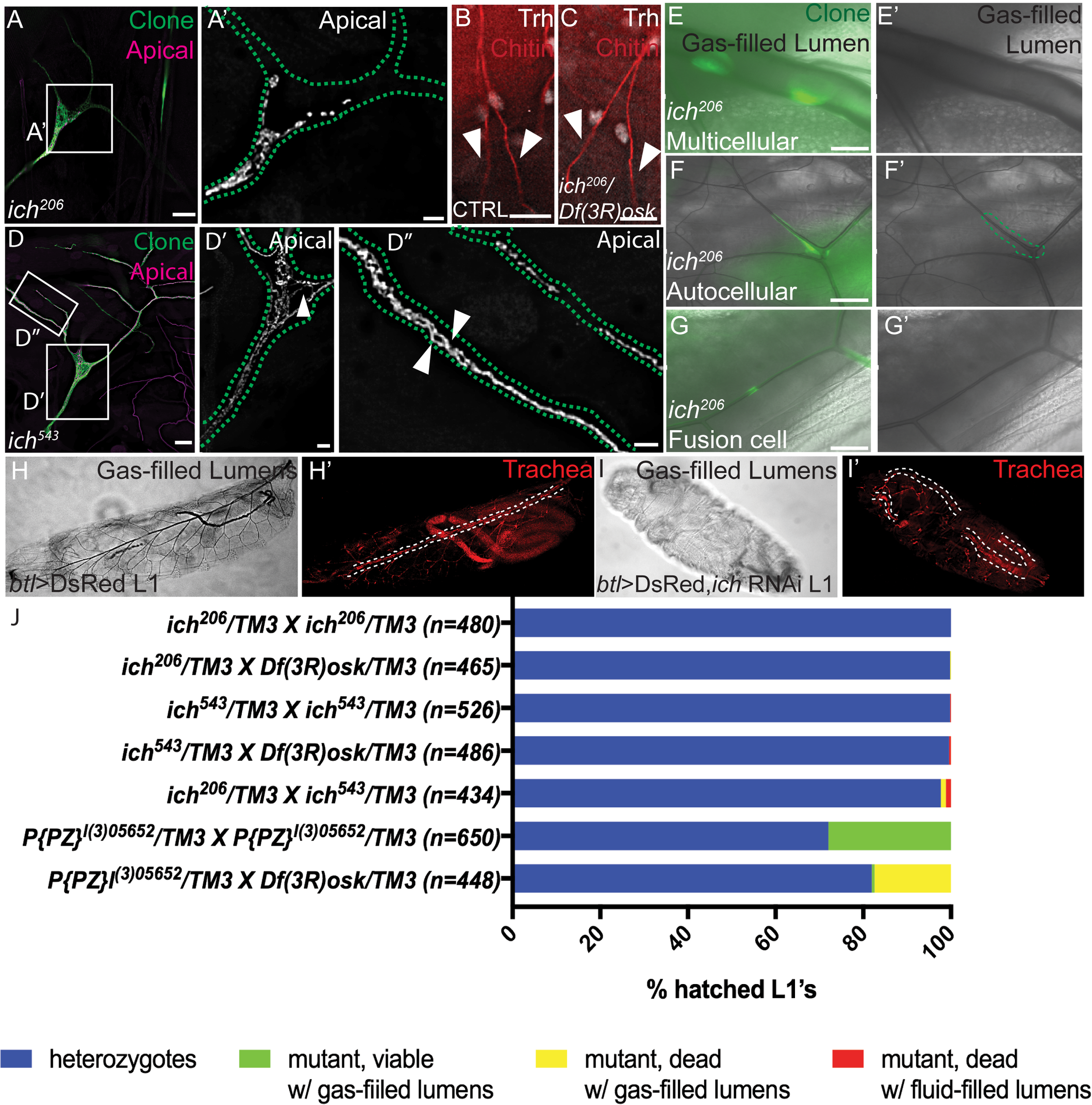

Supplement: S1 Fig — (A-A’) ich206 terminal cell clone exhibiting severe pruning (A) and tube growth defect (A’). (B,C) Wild-type control (C) and ich206/Df(3R)osk (D) embryonic terminal branches visualized by fluorophore-conjugated chitin-binding probe. ich terminal cells are able to extend seamless tubes (arrowheads) beyond the cell body, as marked by the terminal cell nucleus (Trh). Larval (D-D”) ich543 terminal cell clone exhibiting an accumulation of multiple lumens, often with convoluted trajectories (arrowheads in D’, D”). (E-G’) GFP-labeled ich206 MARCM tracheal clones visualized in wholemount heat-killed larvae. Fluorescent images are superimposed on brightfield. Loss of ich in isolated dorsal trunk (E, E’), autocellular (F, F’), and fusion branch (G, G’) cells caused no gas-filling or overt lumen defects. (H-I’) Wholemount heat-killed btl>DsRed (H,H’) and btl>DsRed, ich RNAi (I,I’) first instar larvae (L1). In contrast to wild-type controls (H,H’), btl>DsRed, ich RNAi L1s exhibit pan-tracheal liquid clearance defect (I) as well as a breaks in the dorsal trunks (outlined in I’). (J) Comparison of lethal phase for ich loss-of-function alleles. (Scale Bars: A,D 20 μm; B,C 10 μm; A’,D’, D” 5μm; E-G’, 50 μm). (TIF) [file pgen.1007146.s001.tif]

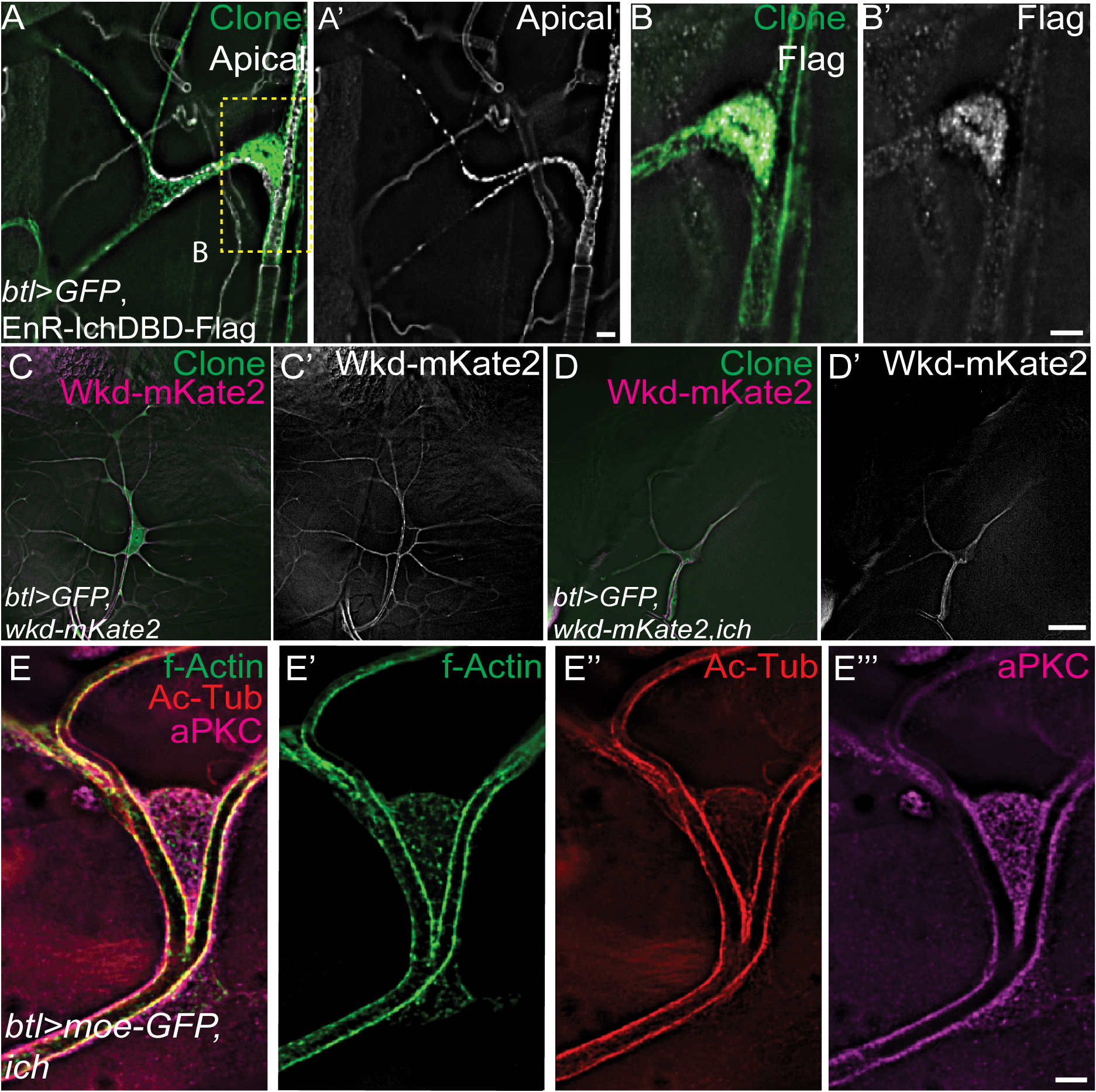

Supplement: S2 Fig — (A-B’) btl>GFP,EnR-IchDBD; 2FRT terminal cell clones stained for GFP, the apical membrane using Wkd antiserum (A’), and the Flag epitope (B,B’). btl>GFP, EnR-IchDBD; 2FRT terminal cell clones exhibit cystic, discontinuous lumens (A,A’). EnR-IchDBD-FLAG localizes to the nucleus of terminal cells (B, B’). (C-D’) Control btl>GFP, Wkd-mKate2; 2FRT (C,C’) and btl>GFP, Wkd-mKate2, ich; 2FRT (D,D’) terminal cell clones overexpressing Ich. In contrast to wild-type controls (C,C’), terminal cells overexpressing full-length Ich (D,D’) exhibit severe pruning with rudimentary lumens (D’). (E-E”‘) btl>moe-GFP, ich; 2FRT terminal cell clones stained to label cortical f-actin (E’), cortical acetylated tubulin (E”), and aPKC (E”‘). Unlike EnR-IchDBD, full-length Ich overexpression in terminal cells does not perturb lumen patency but does disrupt localization of certain apical membrane markers, such as aPKC. (Scale Bar: A-B’, E-E”‘ 5μm; C-D’, 50 μm). (TIF) [file pgen.1007146.s002.tif]

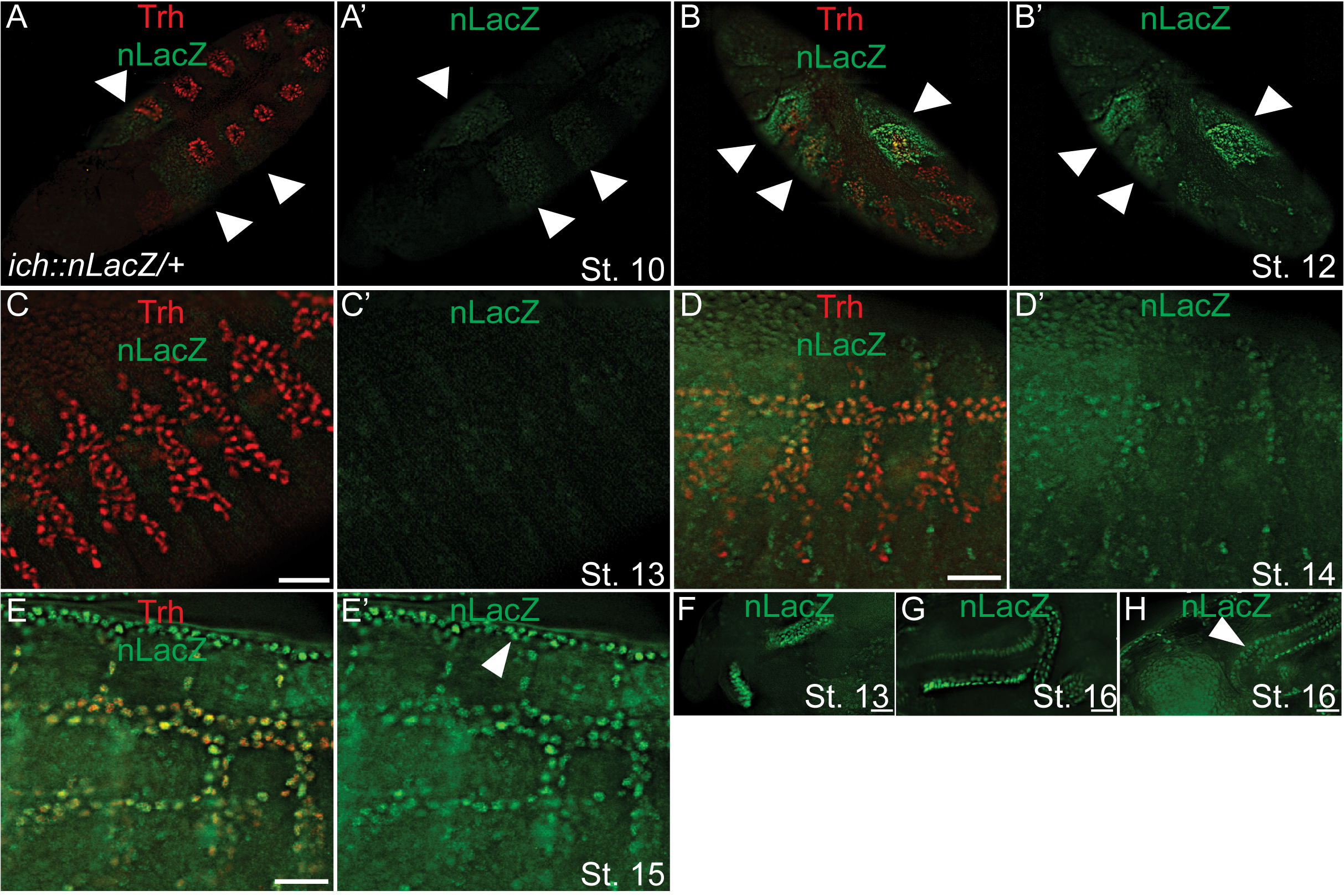

Supplement: S3 Fig — Embryos heterozygous for the ich::nLacZ P element enhancer trap insertion were immunostained for nuclear LacZ (nLacZ, green) and the tracheal specific transcripton factor Trachealess (Trh, red). (A,A’) LacZ signal is first detected in Stage 10 embryos in broad epidermal stripes (A’). During germband retraction (B, B’), epidermal expression is strongest in the T2, T3, and A8 epidermal parasegments (arrowheads in A-B’). LacZ reporter expression is not detected during primary branching (B-C’). Pan-tracheal LacZ expression is first detected at St. 14 (D, D’) and continues during later stages (St. 15: E, E’), coinciding with lumen growth and cuticle deposition. In addition to tracheal expression, LacZ is also expressed in the epidermis (arrowhead in E’), foregut (F, G), and hindgut (arrowhead in H). All are ectodermally-derived epithelia that secrete chitin-based cuticles. (Scale Bars: 20 μm). (TIF) [file pgen.1007146.s003.tif]

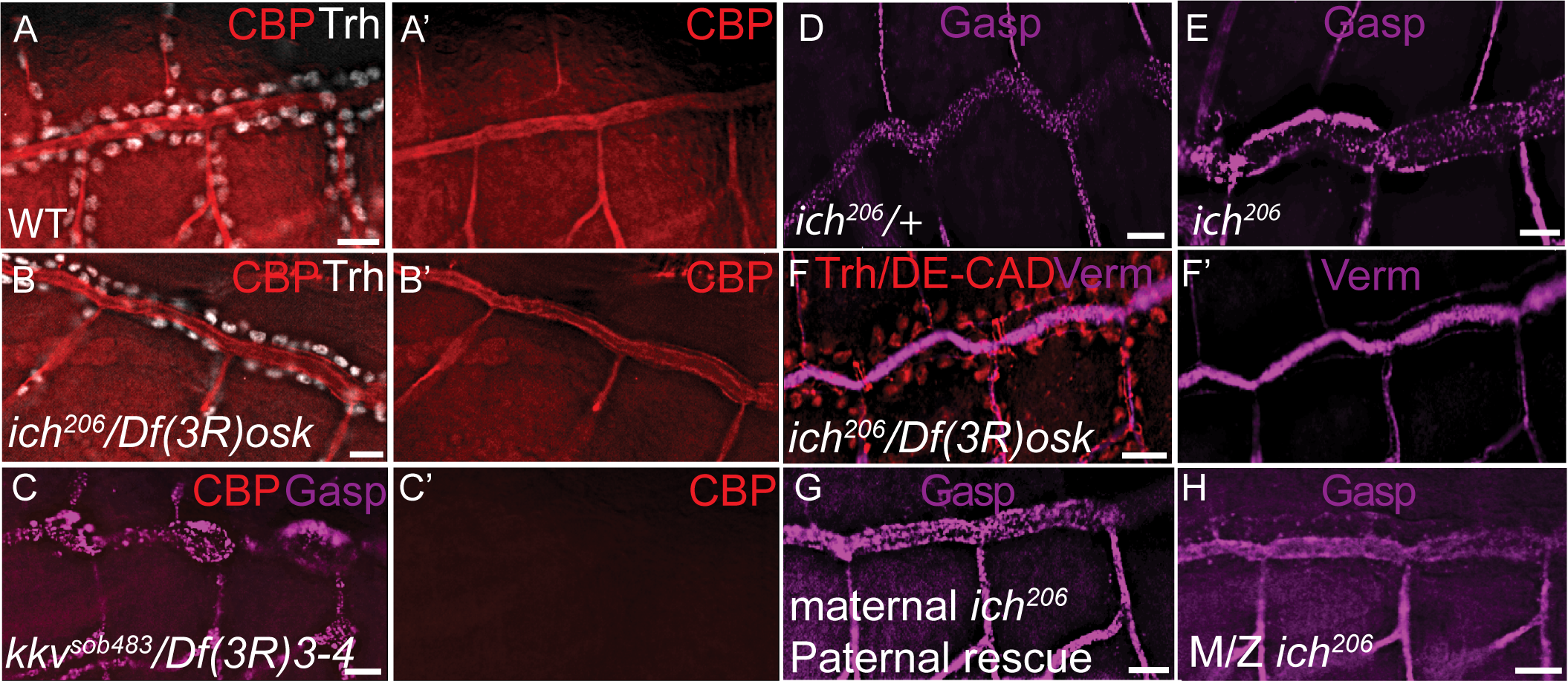

Supplement: S4 Fig — (A-B’) Wild type (WT) (A,A’) and ich206/Df(3R)osk (B,B’) embryos immunostained for Trachealess (white) and chitin-binding probe (CBP, red). ich206/Df(3R)osk embryos deposit a wild-type chitin filament and exhibit neither cystic nor convoluted lumens. (C, C’) kkvsob483/Df(3R)3-4 embryos stained for chitin-binding probe (red) and lumenal matrix protein Gasp (mAb2A12), showing cystic lumen morphology characteristic of chitin-deficient embryos. (D, E) Control (D) and ich206 embryos stained for Gasp, showing ich is dispensable for lumenal accumulation of Gasp. (F-F’) ich206/Df(3R)osk hemizygotes stained for Trh and DE-cadherin (red) and the Chitin Deacetylase Verm (magenta). ich is dispensable for luminal accumulation of Verm. (G,J) Maternal-zygotic ich206 mutant embryos (H) exhibit wild-type lumen morphogenesis in the embryonic trachea. Restoring zygotic ich expression in maternally-deficient embryos (G) has no effect on tracheal lumen morphogenesis. (Scale Bars: 10 μm). (TIF) [file pgen.1007146.s004.tif]

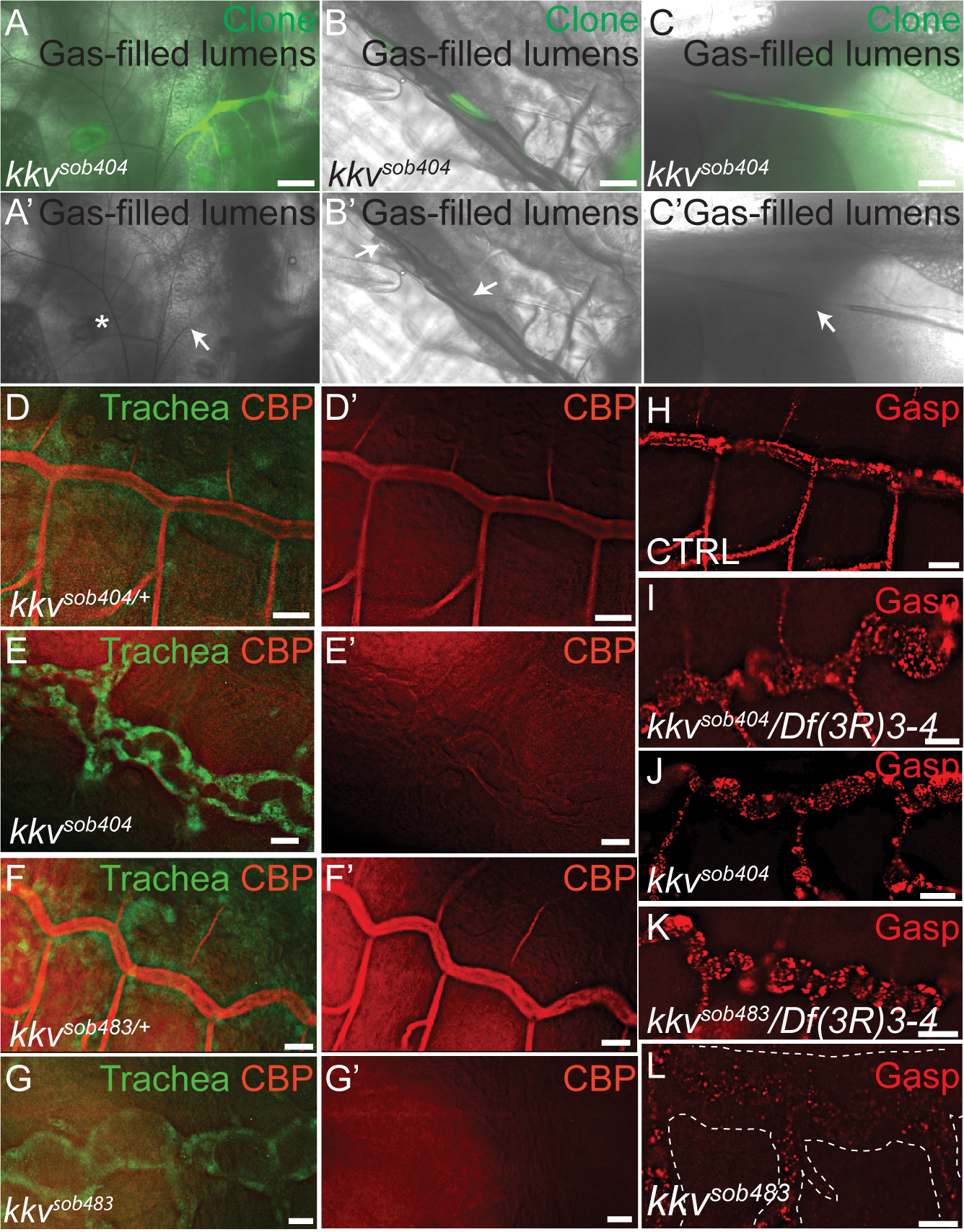

Supplement: S5 Fig — (A-C’) GFP-labeled kkvsob404 MARCM clones in wholemount heat-killed third instar larvae. Unlike wild-type control terminal cells (asterisk in A’), kkvsob404 terminal cell clones exhibit a cell-autonomous gas-filling defect (arrow in A’). Isolated kkvsob404 clones in the dorsal trunk (B, B’) causes cell-autonomous ‘divots’ (arrows in B’) in the gas-filled lumen. Cell-autonomous loss of kkv in autocellular branches (C,C’) causes a cell-autonomous gas-filling defect (arrow in C’). (D-G’) btl>GFP; kkvsob mutant embryos and heterozygous control siblings stained for GFP (green) and chitin-binding probe (red). kkvsob mutants fail to form the transient chitin filament and exhibit cystic lumens in the dorsal trunk. (H-L) Analysis of lumen morphology in wild-type (H), kkvsob hemizygous mutants (I and K), and homoallelic mutants (J and L) using mAb2A12. The cystic dorsal trunks of kkvsob404/Df(3R)3-4 hemizygotes (I) resembles that of kkvsob404 homoallelic mutants (J). However, kkvsob483 homozygotes (L) can exhibit a severe reduction of luminal 2A12 staining not observed in kkvsob483 hemizygotes (K). (Scale Bars: A-C’ 50 μm; D-L, 10 μm). (TIF) [file pgen.1007146.s005.tif]

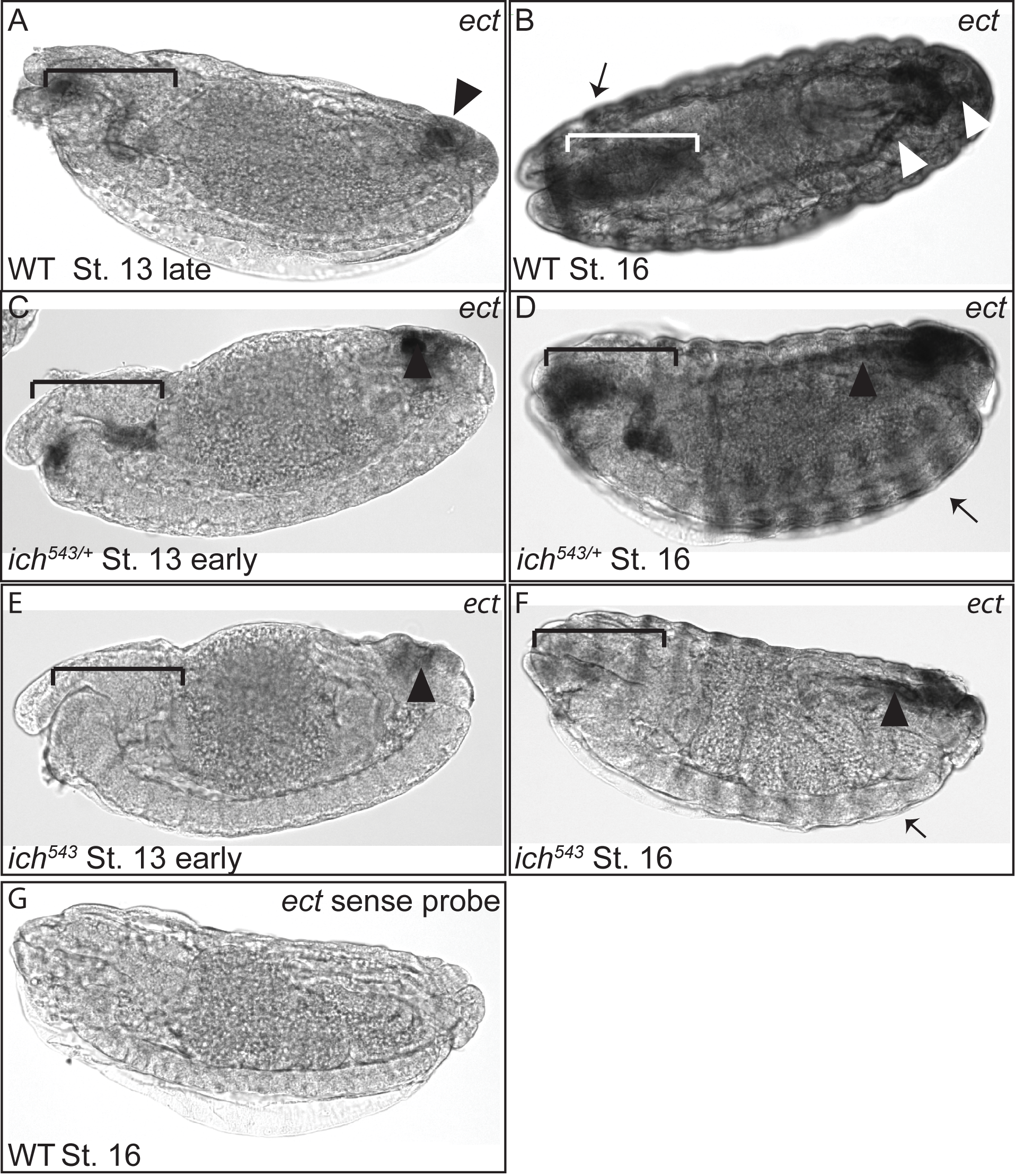

Supplement: S6 Fig — (A, B, G) Wild-type (WT, w1118) embryos hybridized with DIG-labeled anti-sense (A, B) or sense (G) probe. At Stage 13, ect is expressed in the foregut primordium (brackets in A) and posterior spiracles (black arrowhead in A). By Stage 16, ect is expressed in all cuticle-secreting epithelia, including the foregut (bracket in B), epidermis (arrow in B), trachea (black arrowhead in B), and hindgut (white arrowhead in B). This signal is specific to ect transcript because the corresponding sense probe gives no such pattern (G). (C, D) Control ich543/+ heterozygotes hybridized with same anti-sense ect probe exhibit a wild-type expression pattern. By contrast, (E, F), ich543 homozygotes exhibit reduced ect expression in the foregut and epidermis, though ich is not absolutely required for tracheal expression (black arrowheads in E, F). (TIF) [file pgen.1007146.s006.tif]

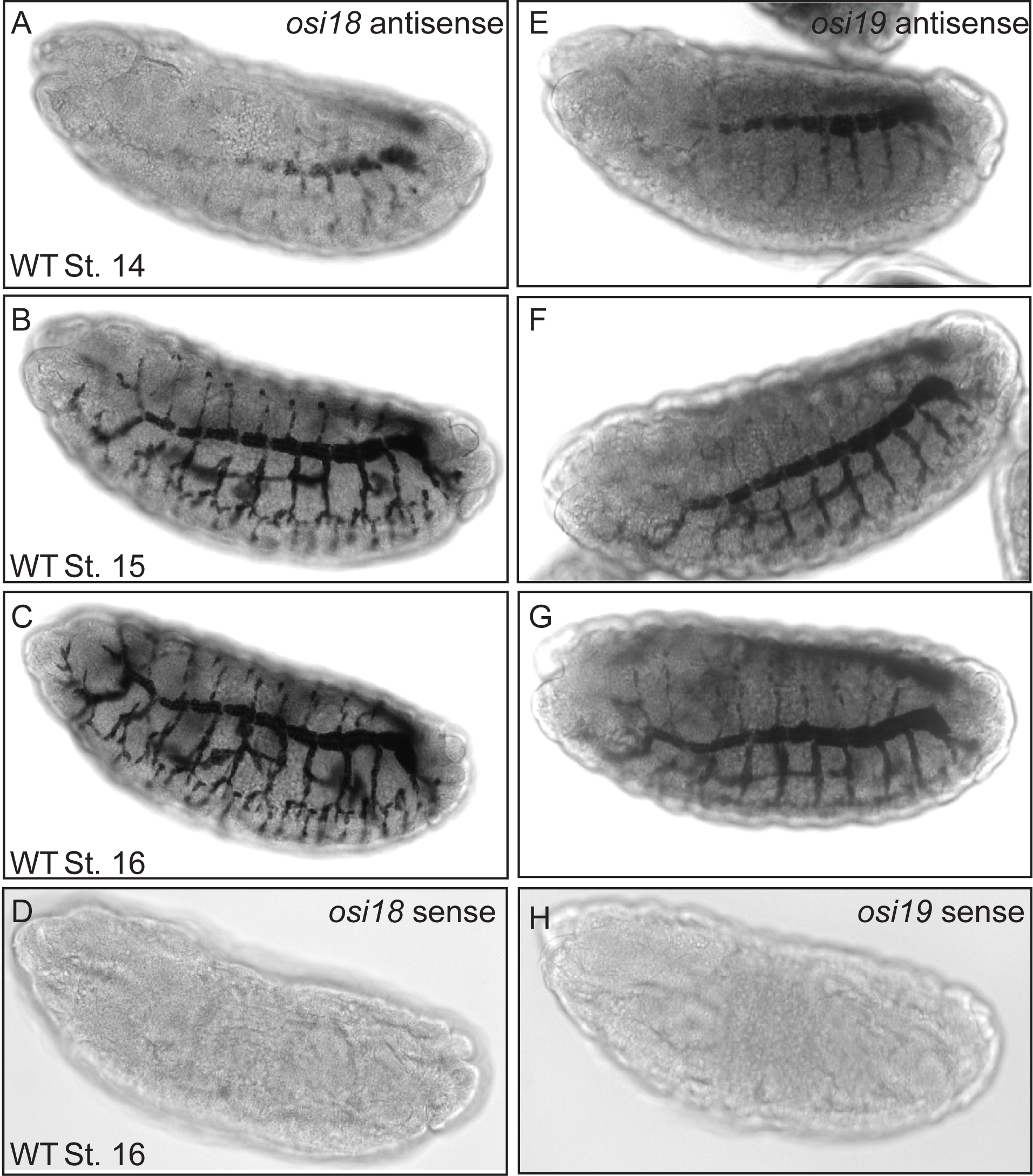

Supplement: S7 Fig — (A-D) Isogenic wild-type (WT,w1118) embryos hybridized with DIG-labeled osi18 (A-C) or osi19 (E-G) antisense probes and corresponding sense probes (D,H). osi18/19 expression is first detected at approximately Stage14 (A,E) in the dorsal trunk and transverse connectives. At Stages 15 (B, F) and 16 (C,G), osi18/19 are strongly expressed throughout the tracheal system. This pattern of expression is specific to osi18/19 transcript because w1118 embryos hybridized with the corresponding sense probes (D, H) give no tracheal signal. (TIF) [file pgen.1007146.s007.tif]

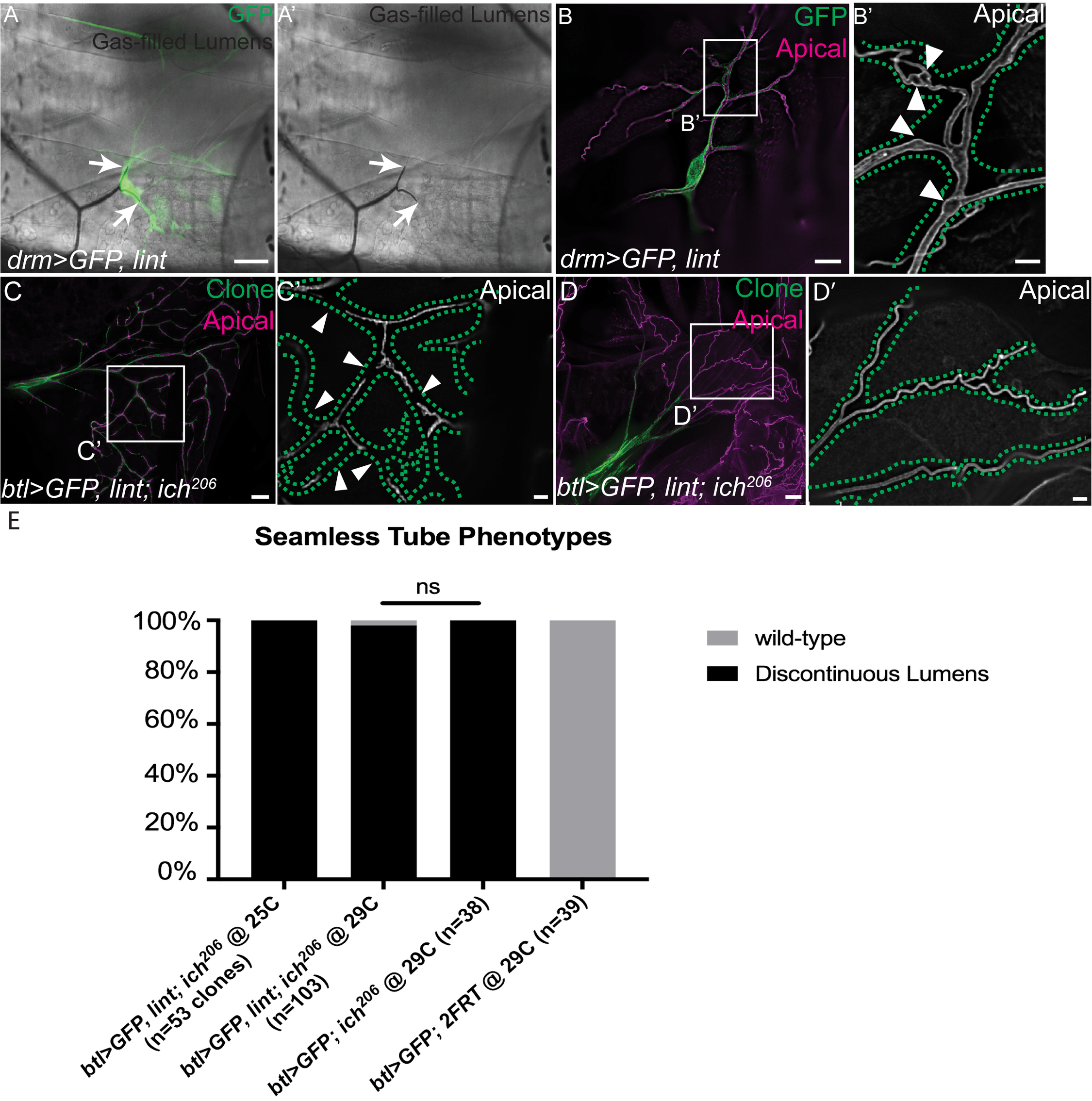

Supplement: S8 Fig — (A,A’) Wholemount images of heat-killed third instar drm>GFP, lint terminal cells overexpressing lint from a UAS promoter. lint overexpression in the terminal cell causes a liquid clearance defect in terminal branches (arrows in A, A’). Unlike terminal cells overexpressing Ich (S2 Fig), lint overexpression (B, B’) does not significantly impair terminal branching or lumen growth (B), but does cause dilations of the apical membrane (arrowheads in B’). (C-E) In most instances, restoring expression of lint in ich206 terminal cells is not sufficient to suppress tube integrity defects (C, C’). However, elevated transgene expression (D,D’) could restore tube integrity at a non-significant frequency(E, 2% of cells, P = 0.5, one-sided Fisher’s exact probability test). (Scale Bars: A, A’ 50 μm; B, C, D 20 μm; B’,C’,D’ 5 μm). (TIF) [file pgen.1007146.s008.tif]

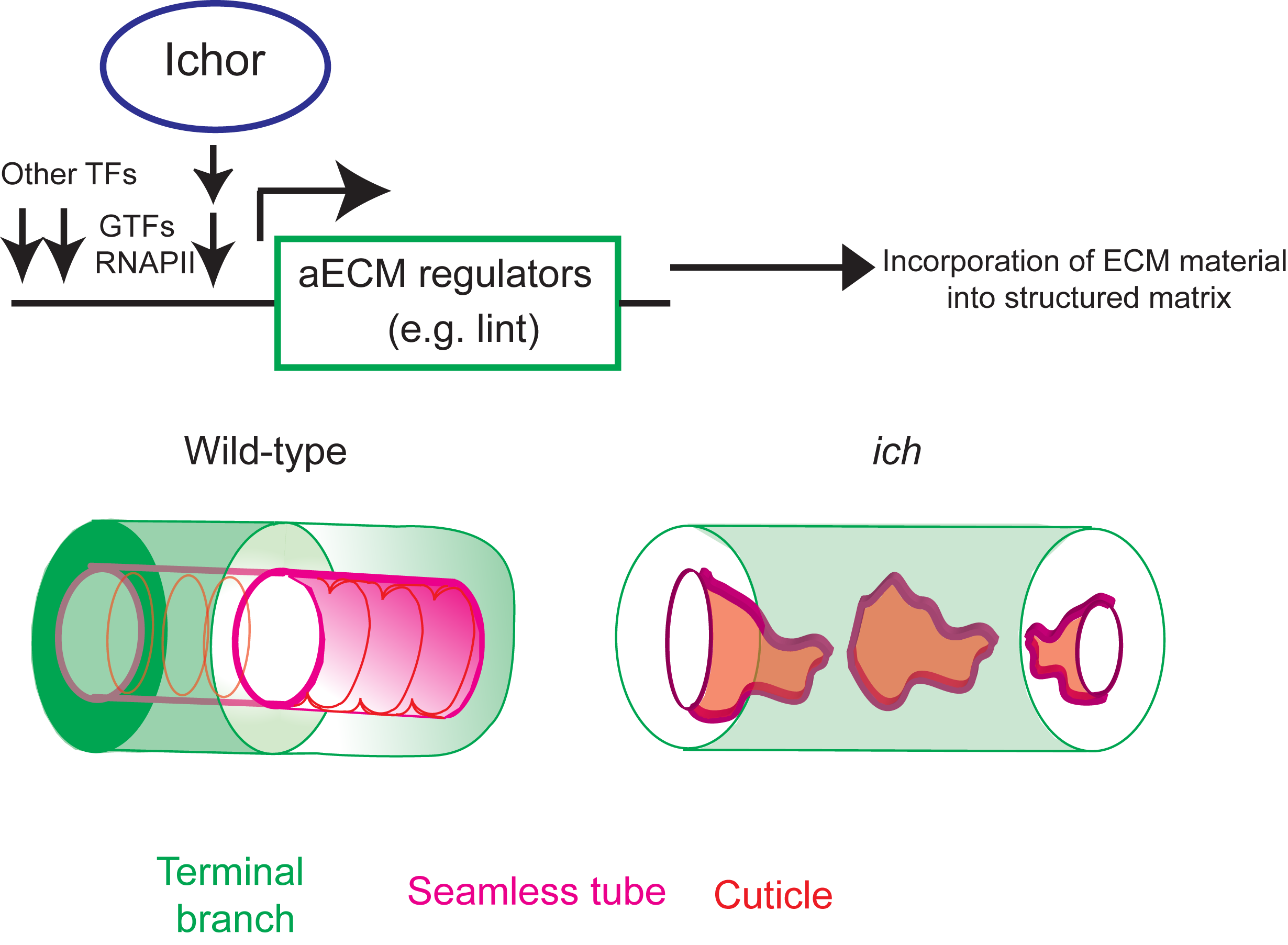

Supplement: S9 Fig — Ichor encodes a zinc finger transcriptional activator (Figs 2 and 4) controlling the assembly of chitin-based cuticles (Figs 3 and S3). In terminal cells, Ichor is dispensable for the secretion of bulk aECM material, but rather, is essential for the organization of this material into an ordered extracellular matrix. In cooperation with other, as yet unidentified transcriptional regulators (compare Fig 6L and 6N), Ichor directly or indirectly activates the expression of known (Fig 7) or putative (Fig 6) regulators of aECM assembly in the trachea. We speculate that Ichor promotes extracellular assembly processes at the lumenal surface of seamless tubes, in part by allowing proteolytic processing events needed for matrix proteins to be incorporated into the terminal cell aECM. The terminal cell aECM is built from a heterogeneous chitinous cuticle organized into a series of taenidial ridges ([63] and Fig 3). When ich function is compromised, taenidiae fail to form and seamless tube lumens are occluded with disorganized matrix material (Fig 3). A lumenal matrix is required in terminal cells for the integrity and shape (Figs 1, 5 and 8) of seamless tubes—perhaps by forming a scaffold to dissipate tension acting on seamless tube lumens, as well as possibly forming an organizing scaffold to coordinate cell hollowing. (TIF) [file pgen.1007146.s009.tif]
